# Optimization of a Transdiagnostic Mobile Emotion Regulation Intervention for University Students: Protocol for a Microrandomized Trial

**DOI:** 10.2196/46603

**Published:** 2023-10-27

**Authors:** Tajda Laure, Rutger C M E Engels, Danielle Remmerswaal, Donna Spruijt-Metz, Stefan Konigorski, Marilisa Boffo

**Affiliations:** 1 Department of Psychology, Education, and Child Studies Erasmus School of Social and Behavioural Sciences Erasmus University of Rotterdam Rotterdam Netherlands; 2 Dornsife Center for Economic & Social Research University of Southern California Los Angeles, CA United States; 3 Keck School of Medicine University of Southern California Los Angeles, CA United States; 4 Department of Psychology University of Southern California Los Angeles, CA United States; 5 Department of Statistics Harvard University Boston, MA United States; 6 Digital Health Center Hasso Plattner Institute for Digital Engineering University of Potsdam Potsdam Germany; 7 Icahn School of Medicine at Mount Sinai Hasso Plattner Institute for Digital Health at Mount Sinai New York, NY United States

**Keywords:** transdiagnostic, mobile intervention, microrandomized trial, mental health, emotion regulation, university students, mobile app, mobile phone

## Abstract

**Background:**

Many university students experience mental health problems such as anxiety and depression. To support their mental health, a transdiagnostic mobile app intervention has been developed. The intervention provides short exercises rooted in various approaches (eg, positive psychology, mindfulness, self-compassion, and acceptance and commitment therapy) that aim to facilitate adaptive emotion regulation (ER) to help students cope with the various stressors they encounter during their time at university.

**Objective:**

The goals of this study are to investigate whether the intervention and its components function as intended and how participants engage with them. In addition, this study aims to monitor changes in distress symptoms and ER skills and identify relevant contextual factors that may moderate the intervention’s impact.

**Methods:**

A sequential explanatory mixed methods design combining a microrandomized trial and semistructured interviews will be used. During the microrandomized trial, students (N=200) will be prompted via the mobile app twice a day for 3 weeks to evaluate their emotional states and complete a randomly assigned intervention (ie, an exercise supporting ER) or a control intervention (ie, a health information snippet). A subsample of participants (21/200, 10.5%) will participate in interviews exploring their user experience with the app and the completed exercises. The primary outcomes will be changes in emotional states and engagement with the intervention (ie, objective and subjective engagement). Objective engagement will be evaluated through log data (eg, exercise completion time). Subjective engagement will be evaluated through exercise likability and helpfulness ratings as well as user experience interviews. The secondary outcomes will include the distal outcomes of the intervention (ie, ER skills and distress symptoms). Finally, the contextual moderators of intervention effectiveness will be explored (eg, the time of day and momentary emotional states).

**Results:**

The study commenced on February 9, 2023, and the data collection was concluded on June 13, 2023. Of the 172 eligible participants, 161 (93.6%) decided to participate. Of these 161 participants, 137 (85.1%) completed the first phase of the study. A subsample of participants (18/172, 10.5%) participated in the user experience interviews. Currently, the data processing and analyses are being conducted.

**Conclusions:**

This study will provide insight into the functioning of the intervention and identify areas for improvement. Furthermore, the findings will shed light on potential changes in the distal outcomes of the intervention (ie, ER skills and distress symptoms), which will be considered when designing a follow-up randomized controlled trial evaluating the full-scale effectiveness of this intervention. Finally, the results and data gathered will be used to design and train a recommendation algorithm that will be integrated into the app linking students to relevant content.

**Trial Registration:**

ClinicalTrials.gov NCT05576883; https://www.clinicaltrials.gov/study/NCT05576883

**International Registered Report Identifier (IRRID):**

DERR1-10.2196/46603

## Introduction

### Background

For young people, the years at university coincide with a transitional period of emerging adulthood [1,2]. This period is not only exciting but also a demanding and uncertain phase in life, and for some, it poses a great risk to their psychological well-being. Globally, it is estimated that between 12% and 50% of all university students are affected by mental health (MH) problems [3-5], the most common ones being anxiety, depression, and substance abuse disorder [3,4,6]. Because of the increased demands for MH support, traditional means of providing it, such as in-person counseling services, are difficult to achieve [7]. Even before the COVID-19 outbreak, which imposed additional challenges to student well-being [8,9], college counseling centers reported that they needed to continually adjust their operations to meet the demands for students’ MH needs [10]. In-person MH support also does not fully fit the needs of all students. In fact, students who experience increased MH problems are especially less likely to seek help, and informal and web-based resources are preferred [11]. Moreover, students who experience higher levels of MH-related stigma are less inclined to use face-to-face MH services and are more open to digital tools such as MH mobile apps [12].

To help students navigate this transitional period of their life and equip them with skills relevant for and beyond university life, it is vital to develop preventive and therapeutic interventions that are easily accessible, can counteract MH-related stigma, and are at the same time cost-effective [13]. As part of a student well-being program introduced at Erasmus University Rotterdam (EUR) in 2019, a mobile app consisting of preventive self-guided MH tools is being developed. Its goal is to help students better manage their MH by supporting them in the development of adaptive emotion regulation (ER) strategies.

Digital tools supporting MH have been proven to be good solutions for existing barriers to care, such as long waiting lines and MH-related stigma, owing to their accessibility and ability to maintain students’ privacy [14-16]. However, there are often substantial problems with the uptake (ie, use and acceptance) and actual effectiveness of these tools. Specifically, if the tool is not used for a recommended period, it is unlikely that it will have the intended effect [17,18]. Low uptake of interventions is often the result of a mismatch between technology and end users’ needs [19]. Technology offers countless possibilities for its use, but if a product is not developed in line with the target user’s needs (eg, values, goals, and abilities) or is not engaging and intuitive to use, the likelihood of such a product being integrated, accepted, and adopted by people is low [14,20]. Therefore, it is important to adopt user-centered eHealth design approaches, where the end user is involved across all stages of the design and development of the intervention as informant or co-designer and has a direct influence on both the outcome and the design process [21]. It is also essential to build a deeper understanding of how an intervention and its components function and under what circumstances they can be most beneficial for different users. This understanding can be helpful to tailor digital interventions to different types of users, optimizing their therapeutic benefits.

This paper describes the protocol for the optimization of a mobile app designed to improve students’ MH, combining a microrandomized trial (MRT) [22,23] with semistructured interviews. The goals of this study are to examine whether the intervention and its separate categories (ie, groups of exercises rooted in different therapeutic approaches supporting ER) have the intended effects and how students engage with and perceive them. In addition, relevant contextual factors, such as emotional state and the time of day, that may have an influence on intervention effectiveness will be explored. The latter will support the development of the prototype of a recommendation system that will connect students to the relevant content in the app based on their needs and characteristics. Ultimately, the study results will support the optimization and further development of this mobile app, which will later be evaluated for its overall effectiveness in a randomized controlled trial (RCT).

### MH Apps for University Students

The current generation of university students, consisting of millennials (those born between 1981 and 1996) and Generation Z (those born between 1997 and 2012), are considered digital natives. Research shows that these generations have developed specific behavioral characteristics owing to the influence of technology on their upbringing; for example, they communicate predominantly through web-based platforms, such as social media networks, because this provides quick and instant delivery of information [24]. This preference is not surprising, given that both young millennials and Generation Z highly value efficiency and the ability to perform multiple tasks at the same time [25]. These generations are digitally connected socially as well as informationally. They grew up in a society where information is instant and easily accessible, which in turn makes them prone to using more flexible learning methods and settings [25]. This explains why learning with the help of internet-based digital systems, such as games or mobile apps, attracts them [26]. Considering this generation’s needs and behavioral characteristics, interventions delivered through mobile phones are a good fit.

Several systematic reviews and meta-analyses have attested that digital interventions have the potential to improve mental well-being among this population [14,15,27-29]. However, most of the scientifically tested digital interventions for this population focus on web-based technologies and less so on mobile technology [14]. Mobile technology enables observation of the immediate context (eg, emotional state, location, and heart rate) through experience sampling and sensors and offers the possibility to recognize an individual’s needs and provide relevant support by using recommendation systems [30]. This permits greater precision in lending support at moments and under circumstances when an intervention is most useful and the person is receptive to it, which can play a crucial role in its uptake [30]. The fit between their needs and the delivered content was indicated to be an especially important feature among university students [18].

Despite the many advantages of digital technology in the field of MH, digital interventions struggle with low uptake and high attrition rates [31]. One of the main challenges is involving and keeping users engaged in the intervention over a longer time span [31,32]; for example, a study examining engagement patterns with trendy MH apps (≥10,000 downloads) found that user retention was only 3.9% two weeks after downloading the app [33]. The low uptake of digital MH interventions is considered one of the most potent barriers to beneficial effects on users’ MH because the intervention cannot have the potential impact when users do not engage with it [13]. Among university students, the main barriers to the use of MH apps include privacy concerns and concerns about the credibility of information. They prefer MH apps that are safe and secure, user friendly, credible, informative, and fitted specifically to their needs [13].

### The Mobile App for Students’ MH

#### Intervention Development

The development of the mobile app was guided by the Center for eHealth Research (CeHRes) road map, an established framework for the development of eHealth tools [19,34] in which evidence and theoretical insights are intertwined with design methods. This road map emphasizes a human-centered participatory design process and iterative approach to eHealth tool development [19]. Following this approach, the mobile app was designed with university students consistently at the center of the process and involved at all stages, from the analysis of the problem and context (ie, contextual inquiry) to the technical operationalization of the intervention. Students acted as informants and co-designers of the intervention. At the early stages of the intervention development, focus groups were conducted with other relevant stakeholders, that is, university psychologists and study advisors who are in close contact with this population. The development process involved a thorough review of scientific literature and multiple rounds of expert sessions involving MH professionals and behavioral scientists to lay a strong evidence- and theory-based foundation for our intervention. In collaboration with serious game and interaction designers, all intervention concepts and designs were tested with end users using qualitative and quantitative methods and iteratively adjusted in line with the results.

#### A Transdiagnostic Approach to MH

The intervention incorporates a transdiagnostic approach to MH (ie, Unified Protocol for Transdiagnostic Treatment of Emotional Disorders [35]) and implements techniques from various therapeutic approaches, such as positive psychology [36], mindfulness [37], acceptance and commitment therapy (ACT) [38], self-compassion [39], and cognitive behavioral therapy [40], to accommodate the diverse needs and content preferences of the student population. Using a transdiagnostic approach implies operating outside the traditional psychodiagnostics boundaries (ie, Diagnostic and Statistical Manual of Mental Disorders, Fifth Edition categories), focusing on the factors that cause or maintain problems that students present with. Instead of restrictedly following protocols aimed at specific disorders, this app will provide evidence-based interventions in a flexible fashion and in line with the needs and preferences of individual students [41].

Targeted psychological factors include negative self-referential thoughts, rumination, experiences of prolonged negative emotional states, and lack of MH literacy. These factors have been highlighted in the scientific literature [35,42-45] as well as by students, university psychologists, and study advisors interviewed at the start of this project. Targeting these transdiagnostic factors has been shown promising in the reduction of depressive and anxiety symptoms [35], both of which are prevalent among the university student population [3,4].

#### Intervention Structure

The current version of the mobile app consists of a suite of MH interventions in the form of short exercises. The goal of these exercises is to teach students how to use adaptive ER strategies such as acceptance, self-soothing, and reappraisal instead of maladaptive strategies such as avoidance, suppression, and rumination. In the short term, this will help them manage their emotional responses to internal (eg, unhelpful thoughts) or external (eg, failing a test) events by improving their ability to adjust or change the intensity and duration of emotional states [46]. In addition, students are frequently prompted to evaluate their emotional states through ecological momentary assessments (EMAs), which enable them to practice self-awareness and self-monitoring. In the long run, improving self-awareness and adopting adaptive ER strategies can work preventively by decreasing the likelihood of developing serious MH problems such as anxiety and mood disorders [47].

#### Our Vision for the Final Version of the App

For the final version of our MH app, we envision that students will be able to access multiple exercises at the same time and in any order and consume the content that is most relevant to their unique MH needs. Such a strategy has been previously proven successful in decreasing stress in university students in an Australian program for students called “thedesk” [48]. In addition, our app will include a recommendation system that will suggest exercises to students considering their unique characteristics, such as their goals, intervention preferences, MH state, momentary emotional state, the time of day, and individual app use history. The recommendation system introduces a potential solution to the low engagement and impact of the mobile intervention because efficiency, system responsiveness, and content relevance to users’ needs are highly valued among this population and have been reported as lacking in available MH apps [18,49].

#### Intervention Optimization Using an MRT Method

To design an adaptive system of interventions responsive to users’ needs, the optimization of their contents and delivery is crucial. This study will combine an MRT with user experience interviews, a novel study design that can provide information on which intervention is effective for whom and in what context [30].

An MRT can evaluate how different intervention components affect an individual in real time (ie, directly after they have used it), and it assesses how this effect varies over time (eg, an intervention may be effective at the beginning, but its effectiveness may decrease with time). In addition, this method can evaluate the moderating role of contextual factors (eg, current mood or the time of day) on the intervention effect, which is especially helpful when optimizing rules for intervention delivery [50,51]. Although it is a young method, an MRT has been successfully applied to evaluate dynamic and user-tailored eHealth interventions focused on weight management [52], smoking cessation [53], and MH [54]. Within the field of MH, NeCamp et al [55] investigated the optimal timing for delivering a mobile MH intervention to medical interns. The authors found that delivering an intervention when individuals experienced lower levels of MH indicated by low mood, poor sleep, and reduced activity was beneficial, whereas intervening when the individuals were doing well had a negative effect [55]. Another MRT study evaluated the impact of an ACT-based mobile intervention on distress symptoms (ie, depressive and stress symptoms) in first-generation university students [56], and the results suggest that interventions prompting mindful awareness and acceptance of internal experience (ie, thoughts, emotions, and physical sensations) were the most impactful for decreasing depressive symptoms. In addition, interventions delivered in the evening were more predictive of decreases in distress symptoms than those delivered in the morning [56]. Both studies signal the importance of considering contextual factors to optimize the impact of digital MH interventions.

Using MRT research design allows for the gathering of more granular and proximal insights about a given intervention, which cannot be captured through traditional RCT designs. In an MRT, unlike in a traditional RCT where individuals are randomized to an intervention or control group at the beginning of the trial, different intervention components (or no intervention) are repeatedly randomized to the participants at prespecified decision points. Thus, in an MRT, the randomization unit is not the participant but the intervention, and participants act as their own controls. Therefore, compared with an RCT, which focuses on between-participant effects, the MRT measures within-participant effects aggregated across participants. The decision point refers to the time of day at which an individual may or may not receive an intervention. Furthermore, the proximal outcome refers to the short-term goals of the intervention (ie, intervention targets) and is seen as the facilitator of the desired distal outcome of the intervention (ie, a mediator of the long-term outcome); for example, if the focus of an intervention is on increasing physical activity, a proximal outcome would be the number of steps taken within 30 minutes after an intervention was delivered, and the distal outcome would be the overall increase in physical activity at the end of the entire intervention period [51].

Finally, owing to repeated randomization, an MRT allows for the evaluation of the moderating role of contextual factors, such as current mood and time, on the proximal effects of the intervention [22].

### Study Objectives

This study combines an MRT design with semistructured user experience interviews to achieve the following research goals.

#### Evaluation of the Proximal Effects of the Intervention

This study will experimentally evaluate whether the intervention as a whole and its separate intervention categories (ie, groups of exercises categorized into 5 therapeutic approaches) have the intended effects on participants’ subsequent emotional states, which, in the long term, may have an impact on students’ MH (eg, depressive and anxiety symptoms) and acquisition of ER skills [47], and whether these effects deteriorate over time. Increases in positive affect and decreases in negative affect when an intervention is received compared with when a control intervention is received are expected and considered effective. For exercises targeting unhelpful thoughts (ie, cognitive defusion [CD]), changes in thought believability and discomfort with the thought before and after the exercises will be observed, and unhelpful thoughts are expected to subside as a result of exercise completion.

#### Evaluation of Intervention Feasibility

The feasibility of the system will be examined by evaluating objective (ie, the pattern of interaction with the exercises) and subjective (ie, participant ratings and experience with the exercises) engagement behavioral patterns.

#### Evaluation of the Distal Outcomes of the Intervention

ER skills and level of distress (ie, stress as well as anxiety and depressive symptoms) will be evaluated before and after the intervention period for the purpose of safety monitoring and to inform the design of a follow-up RCT. In terms of safety, the results will be used to indicate whether negative changes in individuals’ MH (ie, distress symptoms) occurred between baseline and the end of the intervention period. As there is no comparator group, the results will provide only an indication of a potential link between worsened (or improved) distress symptoms and the intervention, which will be further tested in a traditional RCT. In terms of RCT design, given the innovative nature of this intervention, there is no prior data available to estimate its effect size. Therefore, we will use the pre-post evaluation of distal outcomes in this study as a pilot evaluation of changes in the distal outcomes to estimate the potential effect size of the intervention. This estimate will be used to calculate the sample size for the standard RCT, where between-group changes in distal outcomes (ie, ER skills and distress symptoms) will be evaluated.

#### Exploratory Goal: The Impact of Contextual Moderators on Proximal Intervention Effects

The role of the time of day, momentary emotional state, personality type, preferences, and intervention type as moderators will be explored to determine the context under which the intervention and its categories exercises are the most effective.

## Methods

### Study Design

**Figure 1:**
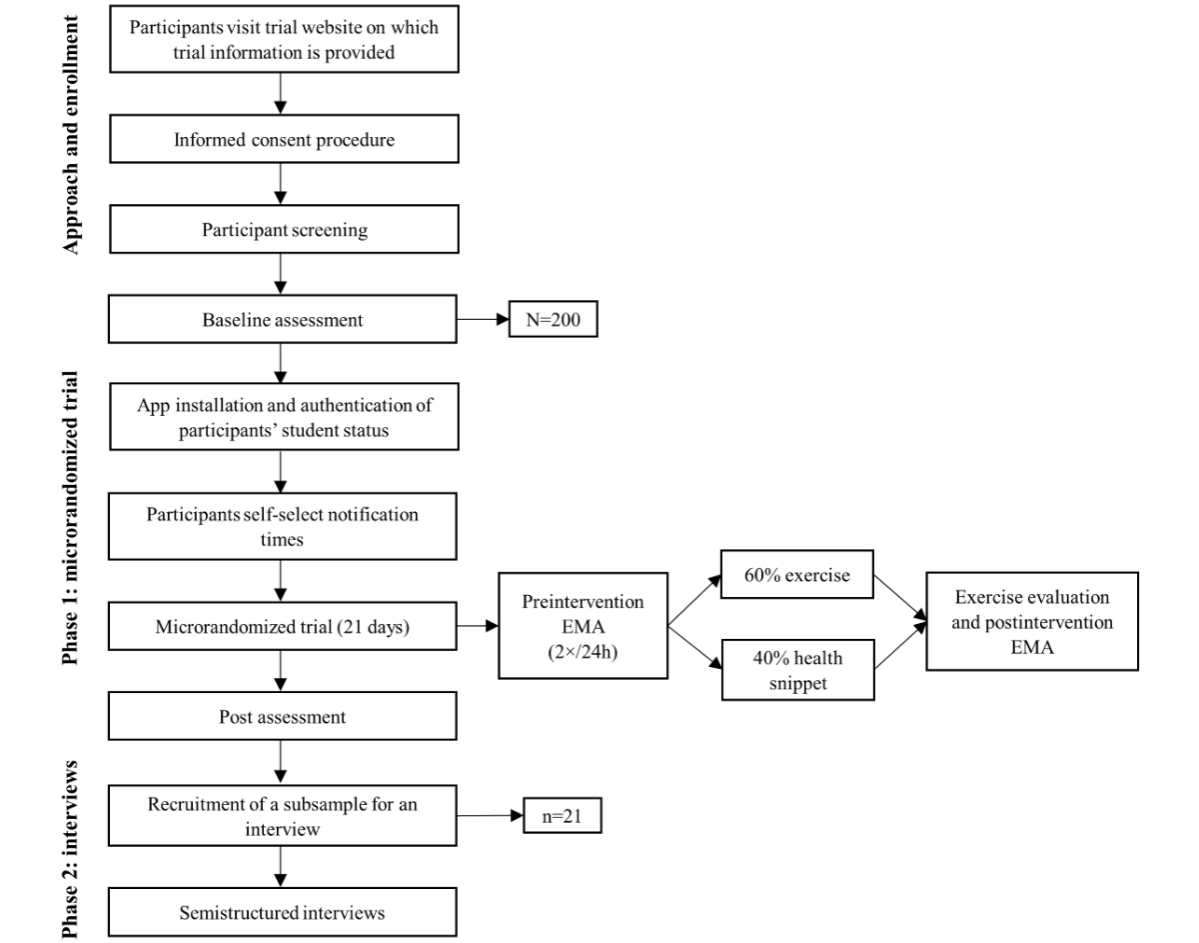
CONSORT (Consolidated Standards of Reporting Trials) flow diagram of participants’ progress through the mixed methods study integrating a microrandomized trial with semistructured interviews. EMA: ecological momentary assessment.

This study uses an explanatory sequential mixed methods approach combining an MRT (phase 1) [22] with follow-up semistructured interviews (phase 2; refer to Figure 1 for an overview). During the MRT, the participants (N=200) will be randomized 2 times per day (morning and evening) to the intervention (ie, an exercise supporting ER; 60% probability) or to a control intervention (ie, a health snippet; 40% probability) over a span of 21 days. Data will be collected at baseline and at the end of the 3-week intervention period, as well as daily via the app before and after the completion of each assigned exercise or health snippet. The primary outcomes include changes in emotional states and engagement patterns with the intervention. For the exercises targeting unhelpful thoughts, differences in thought believability and discomfort before and after the CD exercises will be evaluated. The secondary outcomes include changes in the 4 distal outcomes—ER skills, perceived stress level, and levels of anxiety and depressive symptoms—which will be measured at baseline and at the end of the intervention phase (ie, after 21 days; refer to Table 1 for the measurement schedule).

**Table 1:** Overview of measurements performed during phase 1 (microrandomized trial).

	Day 1	Day 2 to day 22	Day 23
Emotional states		✓	
Thought believability and discomfort^a^		✓	
Demographics	✓		
Experience with MH^b^ apps	✓		
Knowledge of approaches	✓		
Use of MH techniques	✓		✓
Ten-Item Personality Inventory	✓		
General Health Questionnaire-12	✓		
Patient Health Questionnaire-9^c^			✓
Generalized Anxiety Disorder-7	✓		✓
Perceived Stress Scale-14	✓		✓
Emotion Regulation Skills Questionnaire	✓		✓

^a^Thought believability and discomfort are measured only before and after the cognitive defusion exercises.

^b^MH: mental health.

^c^The Patient Health Questionnaire-9 is part of the eligibility screening questionnaire; it is not readministered at baseline.

In phase 2, semistructured interviews will be conducted with a subsample of participants (21/200, 10.5%) investigating their user experience. The results of the qualitative phase will be used to interpret the quantitative results, obtaining a well-rounded picture of how the mobile intervention is functioning and its feasibility.

### Participants

#### Phase 1: MRT

##### Overview

A total of 200 students at EUR will be recruited. A web-based sample size calculator for MRT studies [57,58] was used to calculate the sample size based on the duration of the intervention period (21 days), expected availability intervention randomization probability (60%), and proximal treatment effect. We expect that participants will respond to approximately 70% of the prompts throughout the intervention period (ie, we assume a constant availability of 70%). Furthermore, we assume a linear effect of the intervention on the proximal outcome measures over time with an average size of 0.1 and an initial effect of 0 at the start of the study. In comparison with the large effect size for in-person therapy based on transdiagnostic approaches to emotional disorders [59], we assume a smaller effect size for our study, which is in line with the results of internet interventions for MH in university students [27] and with a similar MRT study protocol examining an ACT intervention [54]. On the basis of these assumptions, 126 participants will generate 80% power to detect the assumed effect with a significance level of .05. We will increase the participant number to 200 to account for a dropout rate of 40%, in line with average attrition rates for MH interventions delivered through smartphones [60].

##### Eligibility Criteria

Participants are eligible to participate in our study if they are currently enrolled at EUR, are aged between 18 and 27 years, own a smartphone with an active phone number, feel comfortable speaking and writing in English, and score between 5 (mild symptoms) and 19 (moderately severe symptoms) on the Patient Health Questionnaire-9 (PHQ-9) [61]. The last criterion was selected to ensure that participants in the study experience a certain degree of MH problems, thereby making the interventions in the app more relevant to them and allowing for the observation of intervention impact within a population that is likely to derive the greatest benefit from it. Participants who have experienced significant suicidal thoughts over the past month; have a medical diagnosis of psychosis or bipolar disorder, severe clinical depression, or anxiety disorder; or are undergoing psychopharmacological treatment or treatment with experimental drugs are ineligible for this study.

#### Phase 2: Semistructured Interviews

From the 200 participants included in the MRT study, 21 (10.5%) will be invited to participate in the semistructured interviews that will occur within 2 weeks of the completion of the MRT study. We expect that 21 participants will provide adequate saturation of themes [62,63]. The subsample will consist of participants with different intervention completion rates (low [<30%], medium [30%-70%], and high [>70%]) to gain a better understanding of engagement patterns and user experiences with the intervention. We aim to balance the sample across gender and study level (ie, bachelor’s or master’s course) using a purposive sampling strategy. Each adherence group would thus consist of 7 participants: 3 (43%) women, 3 (43%) men, and 1 (14%) nonbinary individual. We will attempt to ensure that this subsample comprises roughly equal proportions of bachelor’s students and master’s students. In case thematic saturation is not reached after interviewing 21 participants, we will attempt to recruit additional participants. However, should saturation be achieved before interviewing 21 participants, we may opt to stop recruitment earlier.

### Intervention

#### Overview of the App Content

The intervention under study is delivered through the student well-being app developed by EUR in collaboration with IJsfontein, a media company specializing in serious game design. The version of the app used in this study consists of 3 core features building the intervention flow: (1) EMAs assessing participants’ emotional states before and after engaging in the assigned exercise or control intervention, (2) a visualization of participants’ emotional states acting as immediate feedback to EMA completion, and (3) a total of 20 exercises targeting different ER skills as well as 20 health facts (control intervention; refer to Multimedia Appendix 1 for example intervention pages).

The exercises are categorized into 5 intervention categories that correspond to the 5 therapeutic approaches integrated in this app: *upregulation of positive affect, mindfulness, CD, relaxation and breathing,* and *self-compassion* (refer to Textbox 1 [36, 37, 39, 64] for a list of the exercises). Each therapeutic approach is explained in an article available to participants when they engage in the corresponding exercise. The exercises are relatively short (each lasts between 3 and 12 min) and actionable in the moment. They were selected owing to their potential to directly regulate students’ emotional states and can be completed without extensive exercise background knowledge. Each exercise includes a brief explanation of the rationale behind the exercise as well as debriefing information explaining how the exercise works in general and what the reason might be for experiencing discomfort upon its completion.

**Overview of the exercises classified into 5 intervention categories. The content of the exercises is grounded in the therapeutic principles of positive psychology [36], mindfulness [37], acceptance and commitment therapy [64], yogic breathing, and self-compassion [39].**

**Upregulation of positive affect**

- 3 good things (adapted from Seligman et al [65])
- Savoring the present moment
- Positive self-talk

**Mindfulness**

- 5 senses exercise
- Tracking your thoughts in time (adapted from Hayes and Smith [66])
- Follow your breath (adapted from Hayes and Smith [66])
- Body scan

**Cognitive defusion**

- Milk, milk, milk (adapted from Hayes and Smith [66])
- Bad news radio (adapted from Hayes and Smith [66])
- Labeling (adapted from Hayes and Smith [66])
- Leaves on stream (adapted from Hayes and Smith [66])
- Naming and thanking your mind (adapted from Hayes et al [64] and Harris [67])
- Shape your thought (adapted from Hayes and Smith [66])

**Relaxation and breathing**

- Alternate nostril breathing
- Boxed breathing
- Diaphragmatic breathing
- Progressive muscle relaxation (adapted from Jacobson [68])

**Self-compassion**

- Self-compassion break (adapted from Germer and Neff [69])
- Talking to a friend (adapted from Germer and Neff [69])
- The compassionate friend (adapted from Germer and Neff [69])

#### Intervention Categories

##### Upregulation of Positive Affect

Positive emotions play an important role in people’s well-being. They alleviate the effects of negative emotions that are considered the root of anxiety and depressive disorders and promote resilience and positive MH [70-72]. Several meta-analyses have shown that positive psychology interventions targeting feelings of gratitude, savoring of experiences and sensations, and hope and meaning lead to improved well-being and reduced depressive and anxiety symptoms in both general populations and those considered vulnerable [73-75]. The goal of the exercises in this category is to encourage a focus on positive thoughts and away from negative ones in relation to the current situation or about oneself. These exercises may play an important role in enhancing student well-being because higher levels of positive affect are linked to better psychological and physical health [70,72,76].

##### Mindfulness

Mindfulness refers to present-moment awareness and the acceptance (ie, nonjudgment) of experience independent of one’s emotional state [77,78]. Various studies have shown that being mindful is related to better mental [79,80] and physical [81-83] health in university students. Being in touch with the present moment enables one to react to a stressful situation in an adaptive way by using reappraisal or acceptance [38,77,84] instead of engaging in avoidant and ruminative behaviors. Being mindful helps one to endure a distressing experience by perceiving it as less unpleasant and shifts attention to the here and now rather than to the negative reaction to the current distressing event [85]. Supporting students to practice strategies that will enable them to nonjudgmentally engage with the present moment may help improve their ability to cope and flourish in the academic environment.

##### CD Technique

CD is defined as a process in which people look upon thoughts in a more objective manner and see them as separate from one’s sense of self [86,87]. By contrast, cognitive fusion (the opposite to CD) is a state in which thoughts are perceived as the total truth and thus dominate and regulate one’s behaviors and emotions, potentially leading to maladaptive and inflexible behavioral outcomes [88]. Cognitive fusion is especially maladaptive when people experience negative self-referential thoughts. These types of thoughts are related to elevated psychological distress among students [42] and constitute a risk factor for depression, anxiety [89], and eating disorders [90]. Targeting self-critical thoughts within this population is especially important because many students experience maladaptive perfectionistic tendencies characterized by unrealistic expectations of oneself and problematic evaluations of self-worth and performance [89]. Therefore, the intervention includes exercises that teach different CD techniques, such as deliteralization, labeling, and distancing.

##### Relaxation and Breathing

People can mitigate their emotional states by controlling their breathing [91]. Relaxation and breathing techniques help individuals to decrease their feelings of tension and anxiety, physically and psychologically, and to increase feelings of calmness and evoke relaxation responses that counteract physiological stress responses such as heart palpitations, shortness of breath, and muscle tension. These techniques have a long-standing tradition in psychotherapy and stress management programs [92]. To help students mitigate negative emotional states, such as stress, anger, and tension, the intervention offers breathing and relaxation exercises.

##### Self-Compassion

Self-compassion is considered an effective mood-stabilizing strategy [93]. It refers to the act of being kind, empathic, supportive, and understanding of oneself in moments of hardship. Those practicing self-compassion are less likely to use maladaptive ER strategies such as rumination and suppression and unhelpful behaviors such as self-criticism [94]. Self-compassion is considered a protective factor against academic stress [39,95], homesickness, depression, and problems in the transition to college [96,97]. Teaching students self-compassion techniques may help them stabilize their mood and make space to adaptively cope with the challenges presented by the academic environment.

### Control Intervention

In the control intervention condition, participants receive short snippets of health information about, for example, nutrition, exercise, and sleeping habits. These health-related notions will be phrased in a general, impersonal, and informative fashion. For example, “Did you know that by sneezing the human body is getting rid of infected cells and an average sneeze will spread over 100,000 virus cells up to nine meters? On average, adults catch two to three colds each year. School-age children can have 12 or more colds in a year.” Introducing health information as a control intervention condition will act as a placebo intervention with no effect on ER and keep participants engaged in the postintervention EMA evaluation when they are not randomized to an exercise. The health information might influence participants’ emotional states but to a minimal extent.

### Outcome Measures

#### Primary Outcomes

##### Changes in a Momentary Emotional State

Participants will be asked to evaluate 7 emotional states by answering the question “How [*affect*, eg, happy] do you feel at the moment?” on a scale ranging from 1 (not at all) to 5 (very much). The emotional states are combined into 2 categories of affect: negative (stressed, frustrated, sad, and fatigued) and positive (happy, energetic, and relaxed) [98]. The scores of the emotional states will be averaged per category for preintervention and postintervention EMA assessment. The 2 composite outcomes will be calculated by subtracting the results of the preintervention EMA from those of the postintervention EMA for the positive affect and negative affect categories.

##### Changes in Thought Believability and Discomfort

The CD exercises include a measure of thought believability and discomfort. Participants are asked to assess “How uncomfortable is the thought?” and “How believable (true) is the thought?” on a scale ranging from 1 (not at all uncomfortable or believable) to 100 (very uncomfortable or believable) [99] before and after they engage in the CD exercise.

##### Intervention Engagement

Objective engagement parameters are retrieved from app use analytics (ie, log data) and consist of exercise completion rate, average time spent per exercise, and the times when participants most frequently engaged in an exercise (morning or evening). Subjective engagement parameters of exercise likability (“How much did you like the exercise?”) and helpfulness (“How helpful did you find the exercise?”) scores measured on a scale ranging from 1 (not at all) to 100 (very much) are assessed at the conclusion of each exercise.

##### User Experience Evaluated in Semistructured Interviews

Participants’ user experience with the intervention will be examined qualitatively in follow-up semistructured interviews with a subset of participants (refer to the *Participants* subsection). Students will first be asked to report on their experience with the whole intervention, followed by a discussion focusing on the separate intervention categories and exercises they engaged in during the trial. They will be asked which aspects of these exercises they liked, disliked, or found difficult to complete. Finally, they will be prompted to share what things they would like to see implemented in the further development of the intervention. Apart from this, the interviewees will be asked about their motivations to participate and general experience related to the study participation. The full interview protocol is available in Multimedia Appendix 2. Additional questions may be derived from the evaluation of engagement metrics. In particular, in the interviews, we might pay extra attention to exercises that were evaluated as unhelpful or unlikable to understand why and to figure out how they could be improved.

#### Secondary Outcomes

ER will be measured with the Emotion Regulation Skills Questionnaire [100], which evaluates 8 types of ER skills: awareness, sensation, clarity, understanding acceptance, tolerance, compassionate self-support, readiness to confront distressing situations, and modification of negative emotions. Each skill is assessed with 3 items rated on a scale ranging from 0 (not at all) to 4 (almost always). Apart from the scores on separate subscales, the Emotion Regulation Skills Questionnaire consists of a total score computed as the average of all items. Higher scores indicate higher ER skills.

Symptoms of depression and anxiety are measured with the PHQ-9 [61] and the Generalized Anxiety Disorder-7 (GAD-7) [101] questionnaire, respectively. The PHQ-9 consists of 9 items measuring depressive symptoms, and the GAD-7 is composed of 7 items measuring anxiety symptoms. Both questionnaires instruct participants to indicate how often they have been bothered by different symptoms over the last 2 weeks on a scale ranging from 0 (not at all) to 3 (nearly every day). The summed scores for the PHQ-9 range from 0 to 27 and for the GAD-7 from 0 to 21, with higher scores indicating higher levels of depressive and anxiety symptoms. The PHQ-9 is used both to screen for eligibility and to assess the baseline level of depression.

Stress levels will be assessed with the Perceived Stress Scale-14 [102], which consists of 14 items and instructs participants to indicate how often they felt or thought a certain way over the last month on a scale ranging from 0 (never) to 4 (very often). The summed scores range from 0 to 56, with higher scores indicating higher levels of perceived stress symptoms (refer to Table 1 for an overview of the measurement schedule).

#### Other Measures

Demographics information includes age; gender; university discipline of study (eg, law and medicine); study program (ie, bachelor’s or master’s); prior experience with MH apps (ie, use); and preknowledge and use of, and experience with, the therapeutic approaches included in this study. For the purposes of the recommendation system development, additional information on students’ MH, vulnerability (eg, care duties and functional impairments), and personality traits is collected at the baseline only. These outcomes will be used to explore whether these factors play a role in recommending exercises to students.

Students’ MH is screened with the General Health Questionnaire-12 (GHQ-12) [103]. The GHQ-12 consists of 12 items, each assessing the severity of an MH problem over the past few weeks using a 4-point scale (ranging from 0 to 3). The total scores range from 0 to 36, with higher scores indicating lower psychological well-being.

Personality traits are measured with the Ten-Item Personality Inventory [104], which assesses 5 personality dimensions: extraversion, agreeableness, conscientiousness, emotional stability, and openness to experience. Participants are asked to evaluate to what extent they agree with each statement on a scale ranging from 1 (disagree strongly) to 7 (agree strongly). An example statement is “I see myself extraverted; enthusiastic.”

### Study Procedure

#### Recruitment and Informed Consent Procedures

To secure a diverse sample in terms of gender and students’ university discipline of study, participants will be approached through various pathways. Students who had subscribed to our user research participant pool over the last couple of years will be contacted via email. The study will be advertised on EUR’s campus screens and social media platforms (ie, well-being website and Instagram account); in November 2022, it was advertised during a Student Well-being Week event. Promotional material for the study will be sent to students via communication and marketing offices and student associations.

Interested students will subscribe to the study via a QR code or hyperlink. Those subscribing to the study will be emailed a link to a web page with information for participants and will have 2 weeks to review the information and decide on participation. They will be invited to contact the researcher via phone or email for any study-related question. Students who are interested in participating can indicate this preference at the bottom of the web page and will be automatically directed to the digital informed consent form. Once the informed consent form is signed, participants are screened for eligibility.

#### Phase 1: MRT

Eligible participants will fill in the baseline questionnaires and be asked to install the app on their mobile phones through which the intervention will be delivered over a period of 3 weeks.

Each student will receive a unique randomly generated user ID to access the app to protect their privacy. Participants’ status as EUR students will be verified through the authentication of their student accounts. Once the authentication phase is concluded, participants will be asked to select 1 morning (7 AM to noon) and 1 evening (5 PM to 10 PM) time slot when they believe they are most likely to have time to complete the daily assessments and exercises (Multimedia Appendix 1).

During the intervention phase, participants are asked to complete the intervention flow (ie, preintervention EMA, followed by exercise or health snippet, followed by postintervention EMA) 2 times per day. The prompts (eg, “Good morning! Don’t forget to take a moment for yourself.” or “Psst, did you check your evening exercise for today?”) to complete the flow will be sent to participants at the 2 self-selected time slots. Participants can self-initiate the intervention flow during each time frame by opening the app. If they complete the intervention flow before the self-selected time slot, the reminder for this time slot is skipped. Of note, participants must complete the preintervention EMA to be able to continue and complete the intervention flow. The intervention flow will be available to participants to complete between 7 AM and 2 PM (morning time slot) and between 5 PM and midnight (evening time slot). The completion of tasks in the app is not possible outside of the predetermined time slots. In addition, to ensure that the changes in emotional states are most likely a result of exercise completion rather than other factors, a time limit was set. Participants will have 30 minutes to complete the intervention flow; otherwise, they need to start the intervention flow from the beginning, starting with the preintervention EMA.

Every time participants fill in the preintervention EMA, they have a 60% chance of receiving an exercise and a 40% chance of receiving a health snippet. The 60:40 ratio was chosen to ensure a higher chance of receiving an intervention at every decision point. This strategy has been used in previous MRT studies too [51,52]. For this study, a simple randomization with replacement will be used. The randomization is carried out automatically within the app and is as follows:  randomization of exercise (60% chance) or control intervention; if an exercise is chosen, a second randomization to the intervention categories follows (20% chance for each intervention category), followed by the random selection of 1 exercise within the randomized intervention category (equal chance across exercises); if a control intervention is randomized (40% chance), a second randomization takes place in which a health information snippet is selected. Participants will be asked to complete 42 preintervention EMAs and 42 postintervention EMAs in total during the study. They can receive an exercise 0 times, 1 time, or maximally 2 times in a day. On average, they will be prompted to engage in 1.2 exercises per day and 25.2 exercises throughout the study.

Upon the completion of either the exercise or the control intervention, the participants are asked to reassess their emotional state by completing the postintervention EMA. When assigned to an exercise, in addition to the postintervention EMA, participants will be asked to rate the likability and helpfulness of the exercise. When CD exercises are selected, participants will also complete the corresponding measures of thought believability and discomfort before and after the exercise.

Given that the primary objective of this study is to evaluate the functioning of the intervention rather than observe it in a naturalistic setting, we have implemented an adherence protocol to enhance completion rates and ensure high-quality data. To support participants in staying engaged, they will receive emails or telephone calls from researchers, depending on the number of missed sessions. The adherence protocol is presented in Multimedia Appendix 3.

Immediately after the intervention period is concluded, participants will complete the postintervention assessment, including the secondary outcome measures (refer to Table 1 for an overview of the measurements performed during phase 1 of the study).

#### Phase 2: Semistructured Interviews

In the second phase, semistructured interviews will be conducted with 21 (10.5%) of the 200 study participants. A purposive sampling strategy will be used to balance the sample across gender, study level, and the level of adherence during the MRT. Participants who consented to participate in the user experience interviews will be selected based on the aforementioned characteristics and contacted by the researcher to set up an interview session within 2 weeks of completing the follow-up questionnaire. The interviews will take place on the web or at the EUR behavioral laboratory and will last between 45 and 60 minutes. An overview of the study procedures is presented in Figure 1.

### Ethical Considerations

The study has been approved by the EUR institutional review board (ETH2122-0677) and is registered at ClinicalTrials.gov (NCT05576883).

Participant informed consent will be obtained electronically. The participants will be informed about the study-related goals, procedures, potential benefits and risks, and compensation, as well as privacy and confidentiality aspects. Consenting to participate in interviews and allowing data use beyond the study’s research goals (eg, educational purposes and further research) will be optional, and opting out will not preclude participation in phase 1 of the study.

To protect participants’ privacy and confidentiality, the study procedures as well as research data servers and the app itself comply with the General Data Protection Regulation standards. Research data and personal data will be stored separately. Participants will be assigned unique identifiers, enabling linkage among data collected during the different study phases. Only the primary researchers will have access to the research data and identification key.

Finally, participants in this study will be compensated with digital vouchers for completing the assessments and participating in an interview. To incentivize participants to complete both assessments, the voucher for the baseline assessment is worth €5 (US $5.5), and, for the final assessment, it is worth €15 (US $16.2). The subsample of participants taking part in the semistructured interviews will receive a €10 (US $10.8) voucher to compensate them for their time. Those completing ≥80% of the EMAs during the intervention phase of the study will be included in a lottery, and 4 of them will be randomly selected to win a €50 (US $54.7) voucher each.

### Data Analysis Plan

Preliminary analyses exploring the data set will be conducted, including descriptive statistics and exploratory graphing for all the variables of interest measured during the study, which will allow for the identification of outliers (eg, measurements or recording errors and logical inconsistencies in data). The key baseline variables (eg, sex) will be considered as plausible covariates in the planned analyses.

#### Primary Outcome Analysis

##### Effects of the Intervention on the Proximal Outcomes

The weighted and centered least squares method developed by Boruvka et al [105] to evaluate data from MRT studies will be used to estimate and test the causal excursion effects of the intervention on the proximal outcomes. This method assesses parameters in treatment effect models and allows for the inclusion of covariates that can reduce the variance of the treatment effect estimates. Similar to generalized estimating equations and multilevel models, this method can effectively manage nested data (ie, decision points nested within participants) and within-person dependencies across time in the outcome. A detailed explanation of the analyses is available in Multimedia Appendix 4 [23,51,105,106].

As a primary goal of this study, we will test the overall (average, across all decision points) effect of engaging in any intervention versus engaging in the control intervention on the emotional states measured with the postintervention EMA upon intervention completion. Furthermore, we aim to evaluate whether this effect deteriorates over time by estimating the interaction between the overall intervention effect and the day in the study.

As a subgoal, we will examine differences in the overall effect of engaging in a suggested intervention versus engaging in the control intervention on emotional states for each intervention category separately. We will control for the pretreatment scores of positive and negative affect measured by the preintervention EMA across all models. To account for repeated dependent measures, robust SEs will be calculated using a sandwich estimator, and because we are comparing multiple outcomes, the results will be controlled for multiple testing using Bonferroni correction, that is, we will divide the α value by the number of comparisons being made (ie, 2; hence, we will use α=.025).

Data may be missing if participants do not complete the EMAs before and after an exercise because without the completion of both measurements the observation for the proximal outcome will be missing. If no more than 10% of the data are missing, our primary analysis will be performed as a complete case analysis. If >10% of data are missing, the missing data will be handled by using multiple imputation approaches and outcome models averaged across imputations to adhere to the intention-to-treat principle.

##### Thought Believability and Discomfort Outcome

A multiple linear regression model will be applied to assess the effects of CD exercises on the level of thought believability and discomfort measured before and after CD exercise completion.

#### Feasibility Outcomes

##### Objective Engagement Patterns

Descriptive statistics of engagement metrics (ie, completion rate, average time spent, and the time of day when participants completed the exercises) per exercise and exercise categories will be examined to evaluate exercise performance and inform further design revisions. A linear mixed model will be used to analyze whether there is a relationship between the time of day and exercise completion rates.

##### Subjective Engagement Patterns

To gain insights into subjective engagement with the exercises, we will combine information on (1) the exercise likability and helpfulness ratings and (2) semistructured interviews with a subset of participants purposefully recruited based on their adherence levels (refer to the *Participants* subsection).

##### Exercise Likability and Helpfulness

For every exercise, average scores on likability and helpfulness scales are computed across all participants, with higher scores indicating higher levels of likability and helpfulness of the exercise. Exercises will then be ranked from the most liked and helpful to the least, informing on which exercises may be perceived as difficult by participants and pointing to potential design revisions of exercise content or user experience design.

##### Semistructured Interviews

Information gathered from semistructured interviews with a subsample of study participants will be analyzed using ATLAS.ti software (ATLAS.ti Scientific Software Development GmbH). A multistage standardized thematic analysis will be performed: familiarization with the data, generating preliminary codes, gathering potential themes, reviewing the themes, refining the themes and labeling them, and conducting the write-up of the analysis [107]. Two members of the research team will code the responses for process alignment [108]. In the analysis, we are interested specifically in participants’ experiences with the different types of exercises, in what way these were helpful (or not), and whether participants were able to understand the different approaches and exercises embedded in the intervention (eg, ACT, CD, and self-compassion).

#### Secondary Outcome Analysis

##### Analysis of Distal Outcomes: ER Skills and Distress Symptoms

For estimating the effect of the intervention on participants’ ER skills and levels of distress, we will apply a multiple linear regression model with the following covariates: gender, age, study year, preknowledge of therapeutic approaches, and intervention adherence (ie, number of completed exercises).

##### Analysis of Exploratory Outcome Analysis: Contextual Moderators

For the purposes of optimization of the mobile intervention app, exploratory analyses will be carried out to explore contextual factors, that is, the time of intervention delivery (morning vs evening), momentary emotional state, and vulnerability to MH problems indicated by higher scores on the GHQ-12, as potential moderators of perceived helpfulness and of the proximal effects on changes in emotional states among the intervention and intervention categories. Additional exploratory variables of interest include personality traits and gender. This information will help us form initial decision rules for the algorithm that is being designed for this intervention.

## Results

The study commenced on February 9, 2023, and the data collection was concluded on June 13, 2023. Of the 172 eligible participants, 161 (93.6%) decided to participate. Of these 161 participants, 137 (85.1%) completed the first phase of the study. In the qualitative phase, interviews were conducted with 18 participants: 7 (39%) from the high-adherence group, 7 (39%) from the medium-adherence group, and 4 (22%) from the low-adherence group. Currently, the data processing and analyses are being conducted.

## Discussion

### Overview

This study will investigate the effectiveness and feasibility of digitally delivered therapeutic exercises designed to facilitate adaptive ER among university students. A secondary goal of this study focuses on the evaluation of distal outcomes, that is, changes in ER skills and distress symptoms as a result of the intervention, which will serve as a safety indicator and inform the sample size estimation of the RCT to follow. In addition, contextual moderators that could be relevant for the recommendation system that will link students to exercises relevant to their needs and preferences will be explored.

Compared with other methodologies used for intervention evaluation such as an RCT, the MRT design makes it possible to observe proximal changes in participants’ emotional states directly after they complete an exercise, in addition to requiring fewer participants to achieve sufficient power to detect the proximal main effect of an intervention component [52]. This enables insight into the mechanisms of change (ie, changes in emotional states), elucidating on how the intervention and its components stimulate the desired benefits (ie, increase in ER skills and decrease in depressive and anxiety symptoms). Combining results from the MRT with information on user engagement patterns (ie, log data analytics) and user experience (ie, exercise ratings and user experience interviews) will help unpack the black box typical of studies of digital MH interventions, where often little is known about the mechanisms by which the interventions exert their benefit [109]. The combined results will also indicate intervention components that might need improvements or should be discarded when they play no significant, or possibly impeding, role in facilitating the desired change. This means that if an intervention category is perceived as unhelpful, unlikable, or unclear or was hardly used by participants, and when it was used, it showed a negative or no effect on proximal outcomes, the intervention category (or specific exercises) could be removed from the intervention library or redesigned. Furthermore, the use of a mixed methods approach not only offers the opportunity to gather valuable insight into how the current generation of students perceive and engage with digital MH interventions but also sheds light on how they receive the MRT as a method of evaluation. This information can help optimize future endeavors in the development and evaluation of tools for digital MH interventions. Finally, the results of exploratory analyses and the data set obtained in this study will be used to develop and train the recommendation algorithm, maximizing the likability and usefulness of exercises for users based on individual (ie, personality and MH status) and contextual (eg, the time of day and momentary emotional states) characteristics. Creating a system that can suggest content relevant to its users has been indicated as a top priority for university students to engage and remain engaged in an MH app [18]. Therefore, designing and implementing a recommendation system in the app may be crucial in improving its uptake and effectiveness.

All in all, this study will contribute to the optimization of the intervention content and the system functionalities (ie, recommendation system). An optimized version of the app will be evaluated in an RCT where the full-scale effectiveness on clinical outcomes (eg, ER skills as well as depressive and anxiety symptoms) will be examined.

### Limitations

The results of this study will need to be considered in light of some limitations. First, this study relies heavily on students’ self-reports of emotional states through EMA before and after completing an exercise. Therefore, the participant burden is considered relatively high. However, this was chosen because user research indicated that students do not like to be monitored and consider passive sensing tracking (eg, collection of location and acceleration data) as too invasive. This is in line with studies examining the acceptability of sensing technology among university students [110,111].

Second, in this study, participants are compensated for completing the baseline and follow-up assessments and for participating in the interview (when applicable). In addition, they are incentivized to complete the daily assessment by the prospect of inclusion in a lottery for a €50 (US $54.7) voucher when they achieve a completion rate of ≥80%. Therefore, this design only allows the evaluation of certain aspects of engagement, such as how participants interacted with the exercises and the average likability and helpfulness ratings. We cannot draw any firm conclusions about the actual engagement level with the intervention when used freely in a naturalistic setting.

Third, our mobile app combines various types of exercises based on different therapeutic approaches ranging from self-compassion to positive psychology. The exercises were designed in such a way that they can be completed in the moment, without users’ preknowledge of the approach. In this study, we offer them materials educating them about the underlying exercise approach, but this is kept optional. There is a possibility that some exercises might be more effective when presented in combination with psychoeducational material. In addition, given the randomized nature of the exercises, participants might find some suggestions less appropriate when reporting certain states (eg, recommending an exercise belonging to the upregulation of positive affect category when participants report a high-stress state). However, the experimental goal of the study is to empirically examine how the exercises affect participants’ emotional states and participants’ reactions to the exercises (whether they like them or find them helpful) instead of drawing conclusions based on current psychological theories and our hunches. Current psychological theories seem to be insufficient to inform the development of mobile interventions that include high levels of interactivity and flexibility. This is because prevailing behavior change theories are still based on data providing information on static snapshots of behavior (ie, the predictions of behavior are based on pre-post evaluations) rather than accounting for contextualized and transitory changes in mood states and behavior at the granular level [112-114]. However, an MRT can address the limitations of traditional research methods, such as RCTs, which have shaped current behavior change theories. Specifically, an MRT offers a deeper understanding of the dynamics involved in health behaviors and behavior change, which are influenced by a myriad of contextual factors, including emotional states, physical context, social norms, and expectations [115]. These factors seem to play a role in intervention functioning [51,55,56]. Therefore, with this study, we hope to gather insights and draw experimentally informed conclusions regarding which intervention option is appropriate for whom and in what context.

### Conclusions

In sum, the results of this study will inform further development of the content and system of the digital MH tool that is being developed for university students. The optimization method will help us optimize our mobile app and shed light on how different therapeutic approaches work and why. Such information will hopefully be valuable to the development of modern theories of behavior change because this design can provide information on how different therapeutic exercises influence ER in near time.

## Appendix

Multimedia Appendix 1 Examples of intervention pages of the app (called “Room”).

Multimedia Appendix 2 Protocol for the semistructured interview.

Multimedia Appendix 3 Adherence protocol.

Multimedia Appendix 4 The analytic method used for microrandomized trial data analysis.

## Abbreviations

ACT: acceptance and commitment therapy
CD: cognitive defusion
CeHRes: Center for eHealth Research
EMA: ecological momentary assessment
ER: emotion regulation
EUR: Erasmus University Rotterdam
GAD-7: Generalized Anxiety Disorder-7
GHQ-12: General Health Questionnaire-12
MH: mental health
MRT: microrandomized trial
PHQ-9: Patient Health Questionnaire-9
RCT: randomized controlled trial

## Data Availability

The data collected in phase 1 of the study will be available in the Open Science Framework (OSF) data repository [116].
