# Supplementary material for: Optimization of a Transdiagnostic Mobile Emotion Regulation Intervention for University Students: Protocol for a Microrandomized Trial
Source: JMIR Res Protoc. 2023 Oct 27;12:e46603. doi: 10.2196/46603 (PMC10638637; doi:10.2196/46603)

**
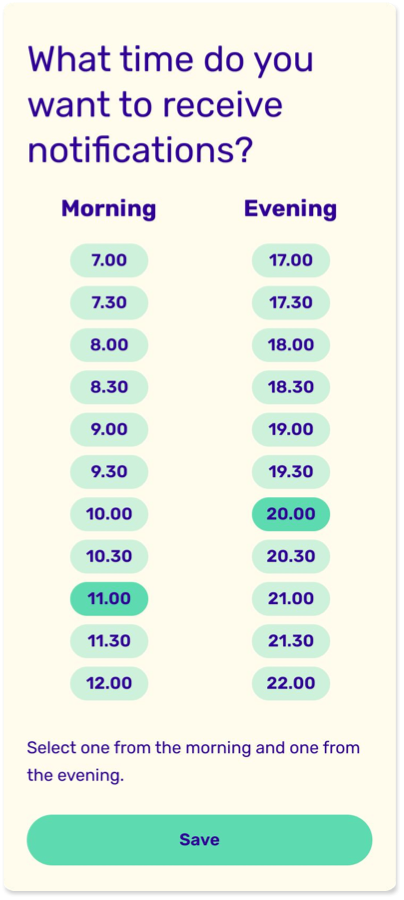
Onboarding screens**


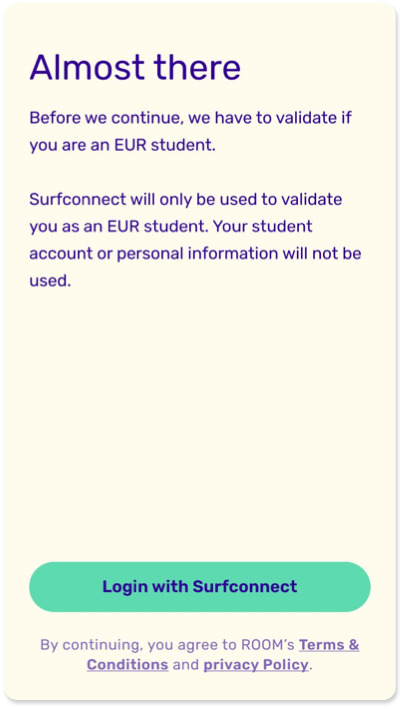

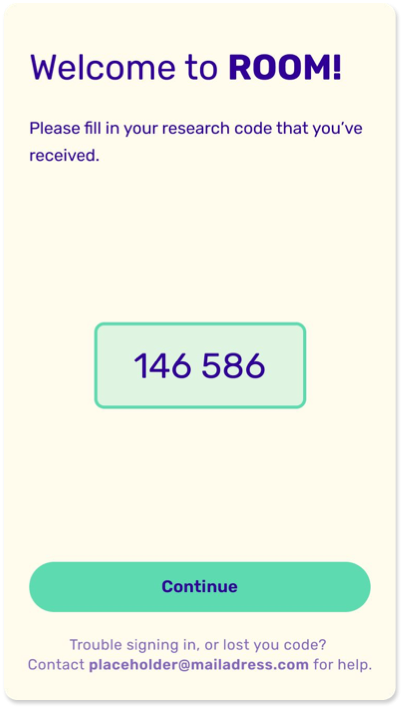

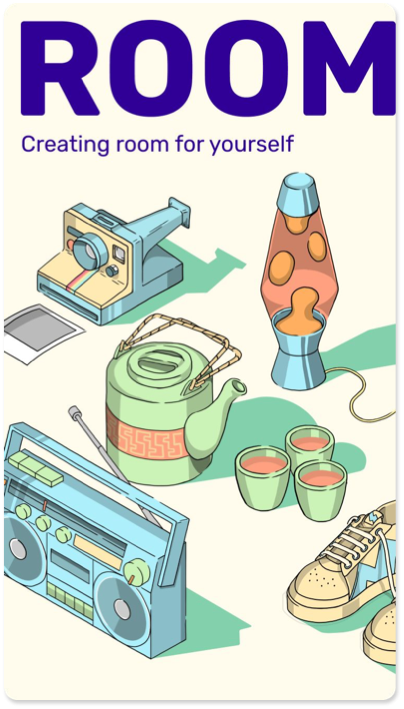


**Example of a reminder, time limit and time window notification**

iv. Time slot selection

Image ii. and iii. depict pages where participant enters unique user ID and verifies their student status through Surfconnect.
Image iv. depicts the page where the participant selects the two time points (one in the morning, and one in the evening) when they are most likely available to complete the intervention flows.

iii. Student status verification

ii. Unique user ID

i. Room app’s cover page

v. App’s home page visible when a participant enters the app and hasn’t yet completed the intervention flow during the morning time slot.


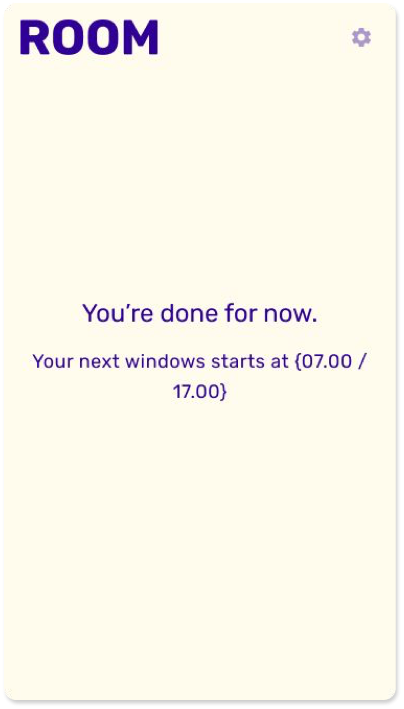

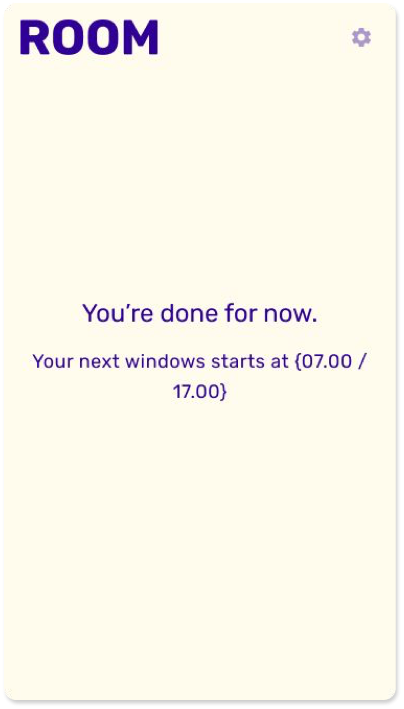

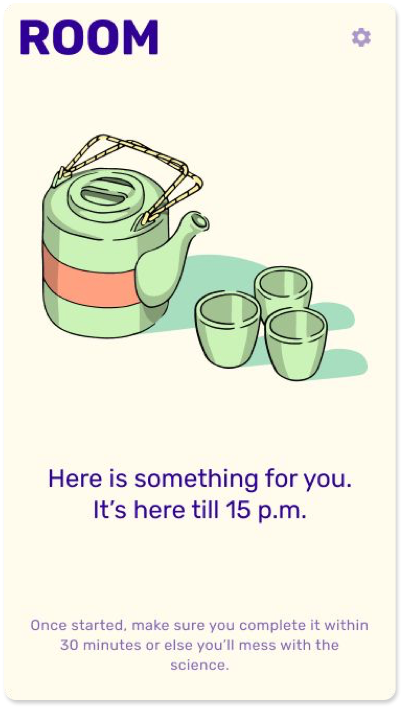

vi. Page notifying a participant they exceeded the time limit to complete the intervention flow.

vii. App’s Home page notifying a participant they have completed the intervention flow. This same page is also shown when participants cannot engage in the intervention (i.e., between 14.00 and 17.00 and 00.00 and 7.00)

**An example of pre and post Ecological Momentary Assessment [EMA]**

Below you can see an example of one out of 7 EMA items evaluating participant’s emotional state. The size of the circle corresponds to the extent (not at all vs extremely) the participant reports to experience certain emotion. Visualization of participants’ emotional states acting as immediate feedback to completion of the EMA.


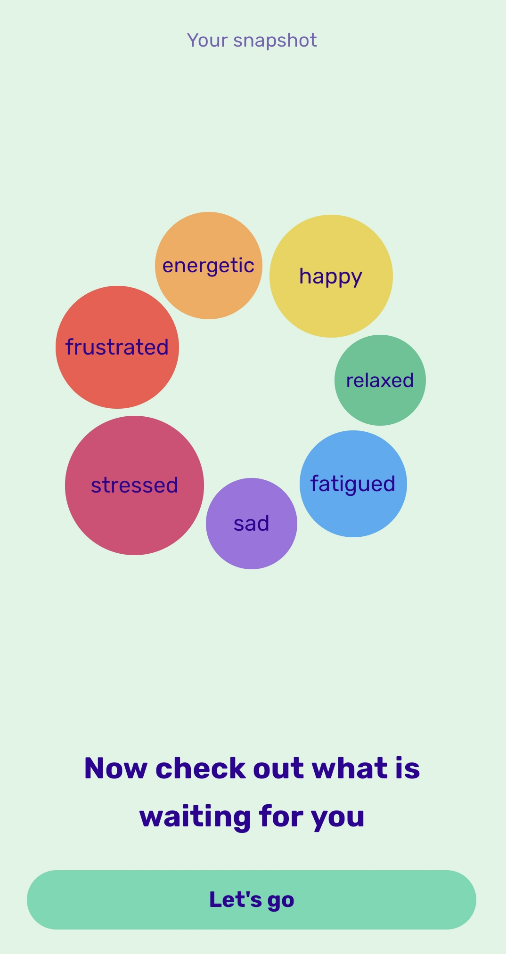

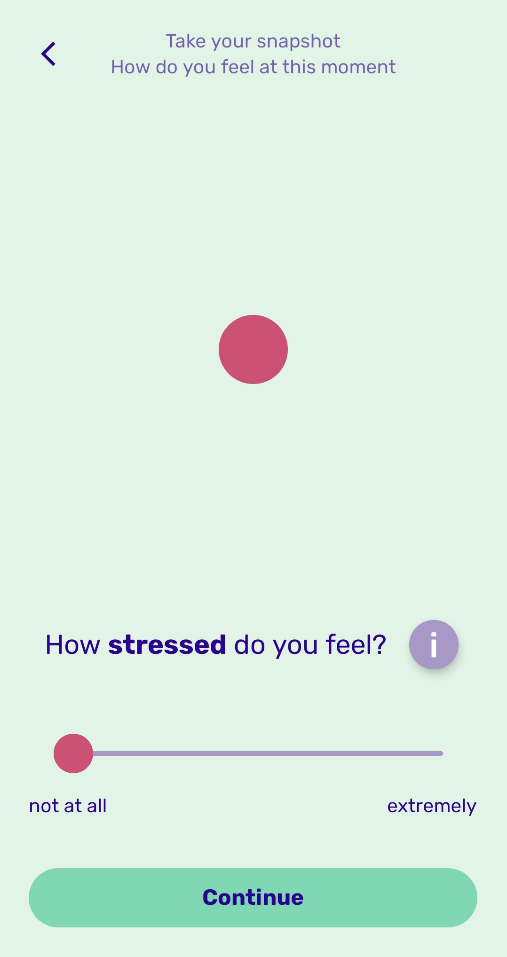

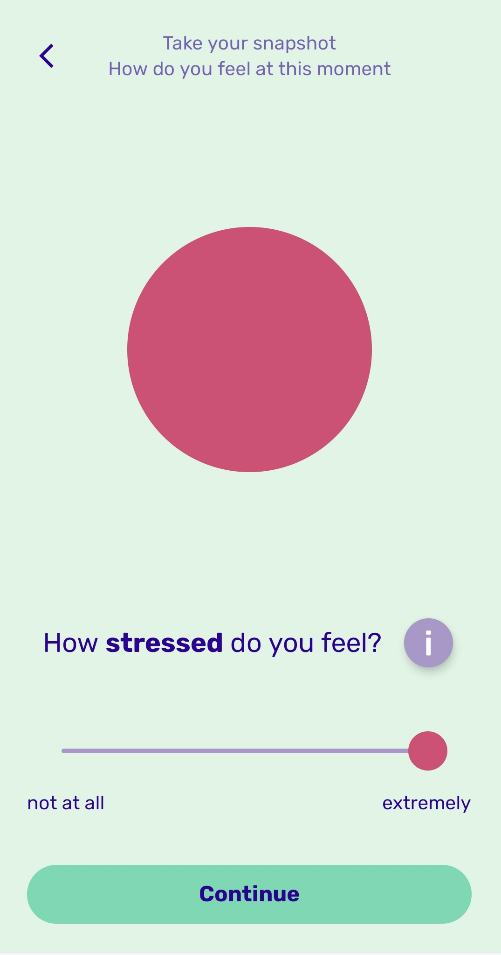

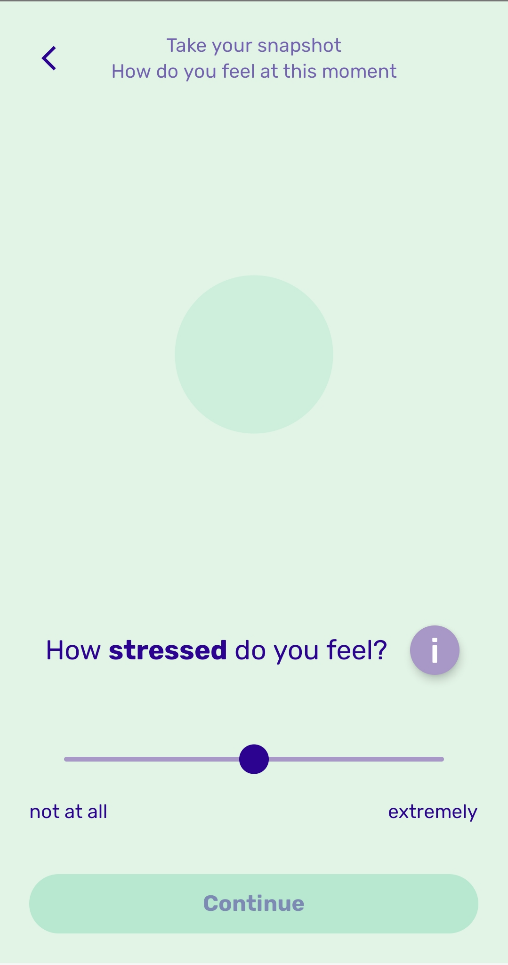


**An example of an exercise flow**

The intervention pages presented on pages 4 and 5 depict a flow of an exercise called *Three good things* (Category: *upregulation of positive affect*). The screens with the teapot and cups are the main screens all participants go through. The purple screens include additional information participants can access if they wish to know more about the exercise (i.e., the purple screen on page 4) or need additional prompts to complete the exercise (i.e., the purple screen on page 5). The page with background information about the exercise is accessed by clicking the icon in the upper left corner. The prompt page is accessed by clicking the info icon positioned in the upper right corner of the green box with the instruction text


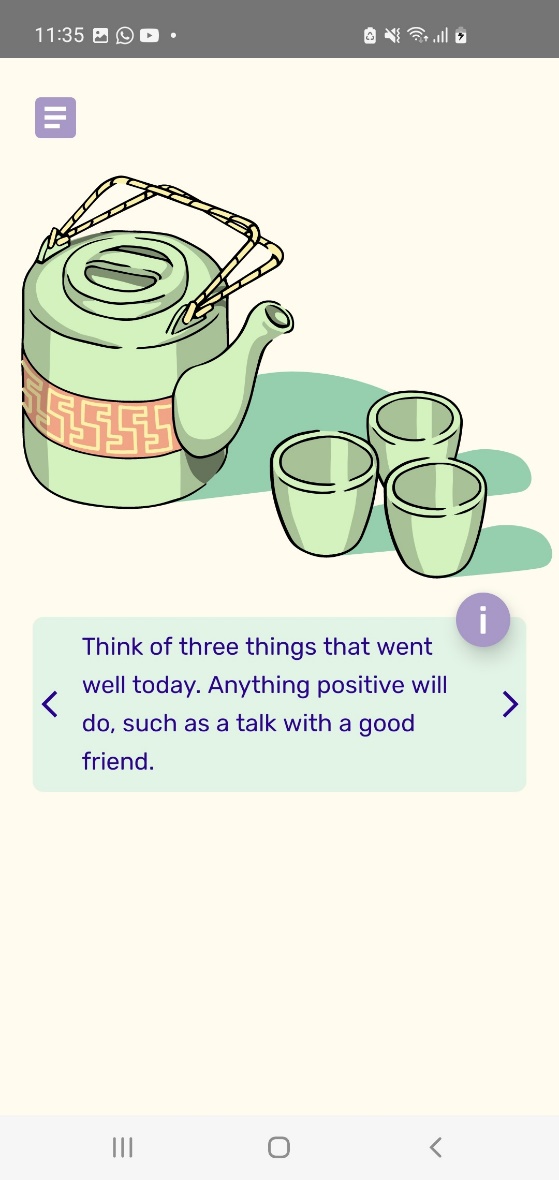
**
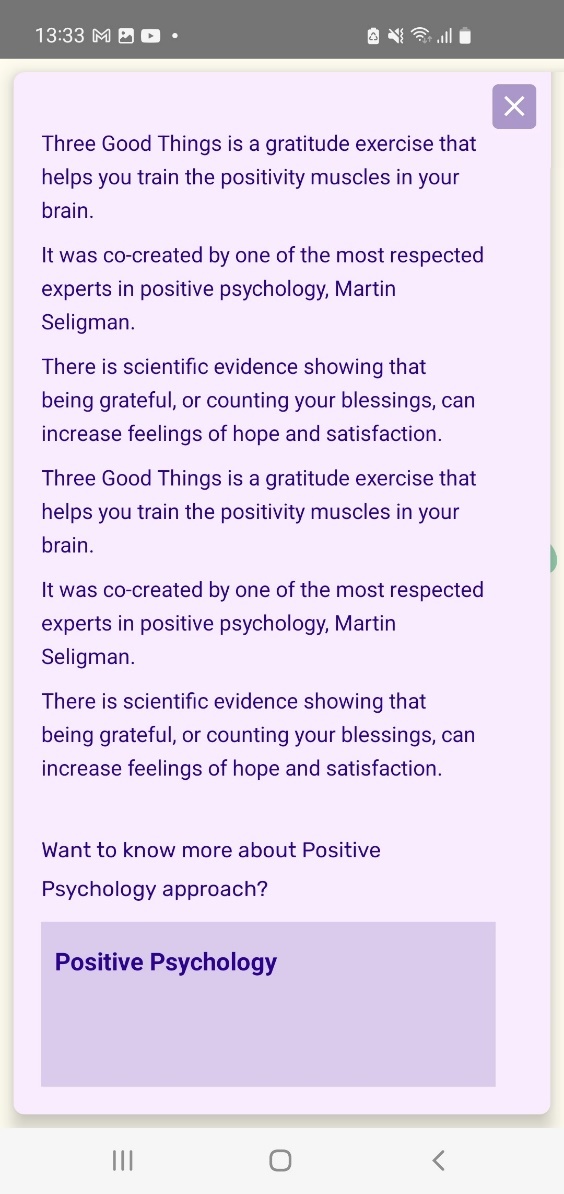

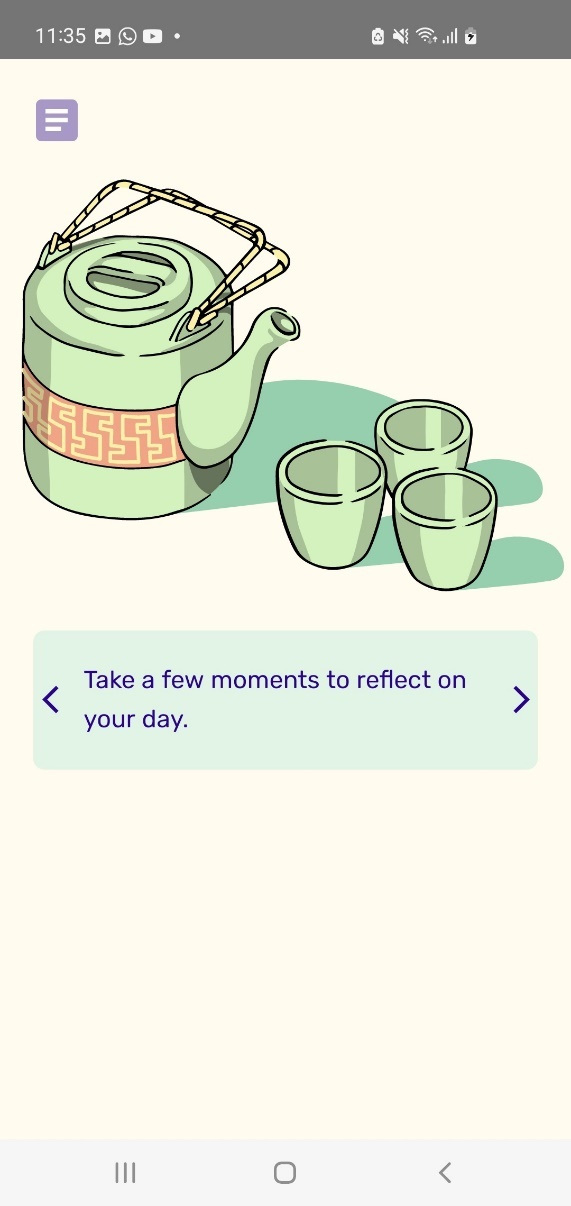

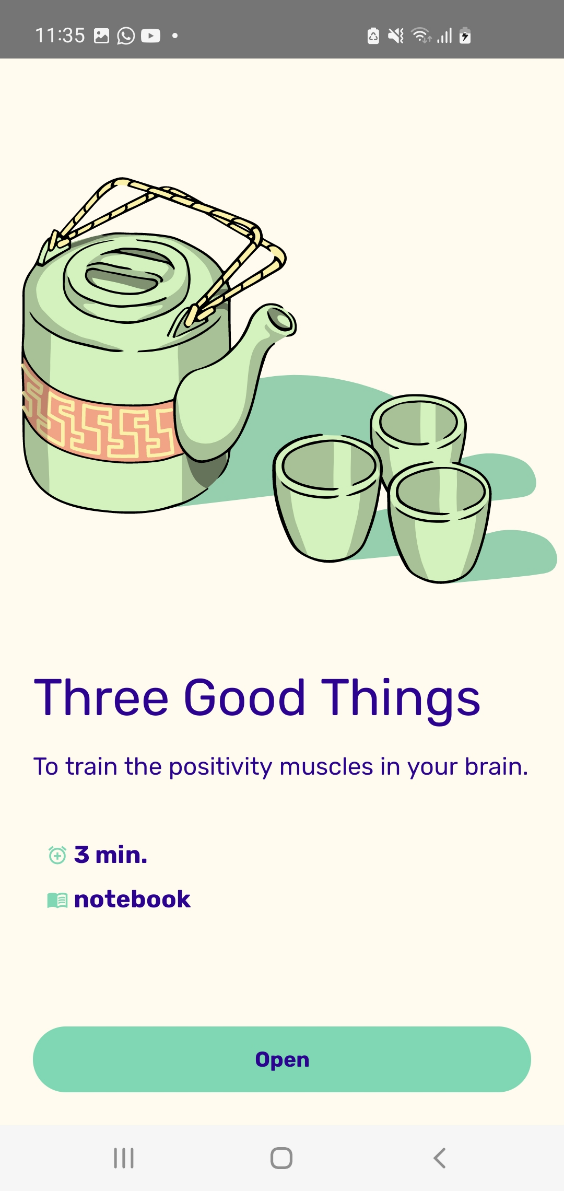
**

**
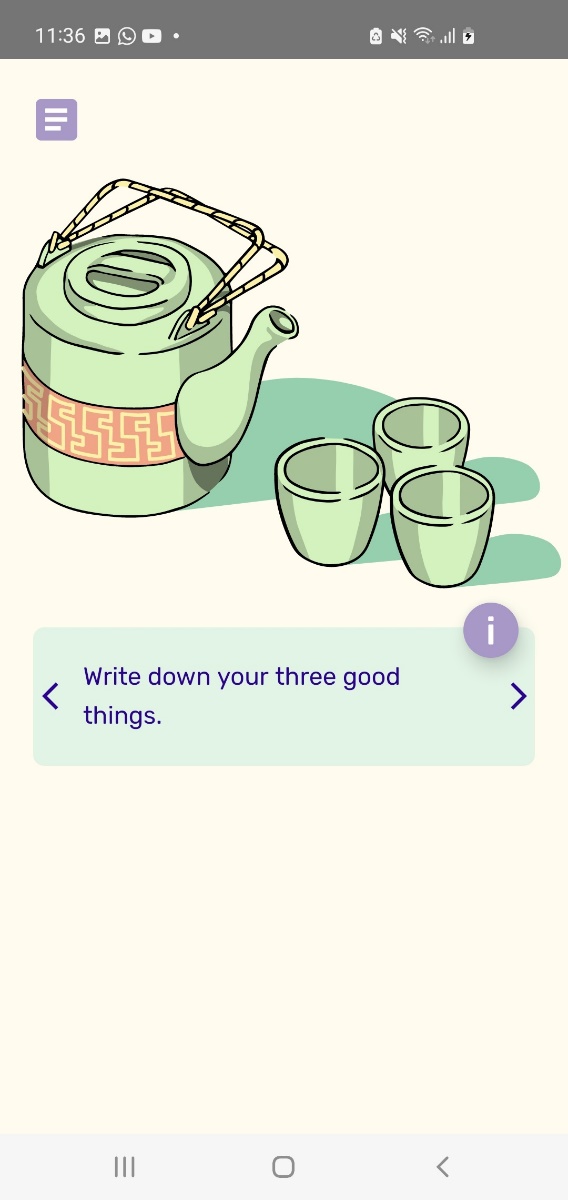

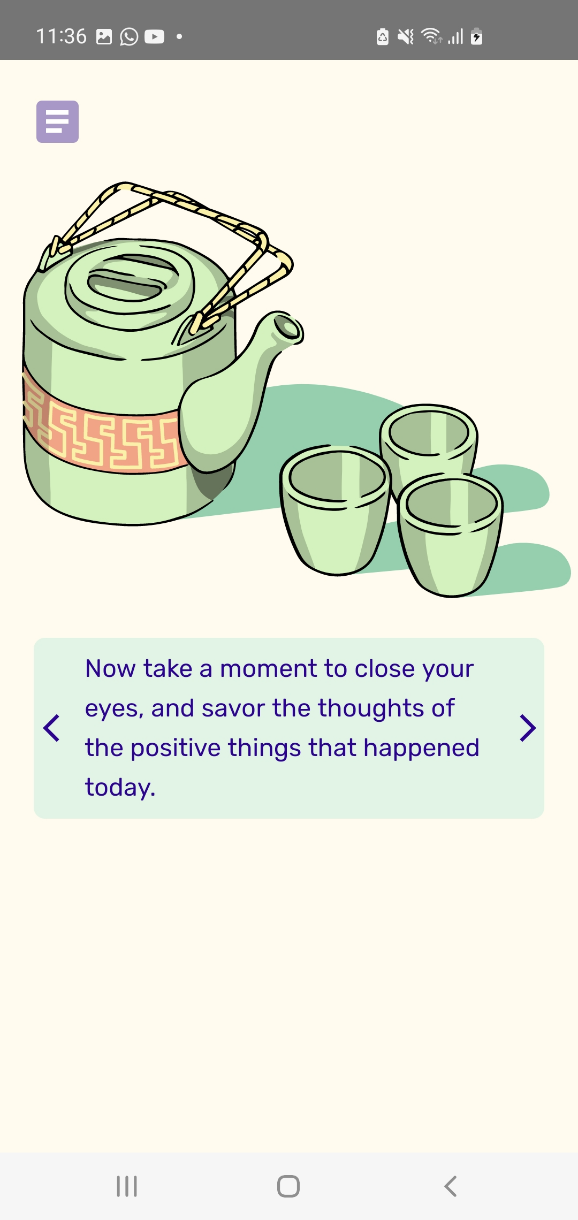
**
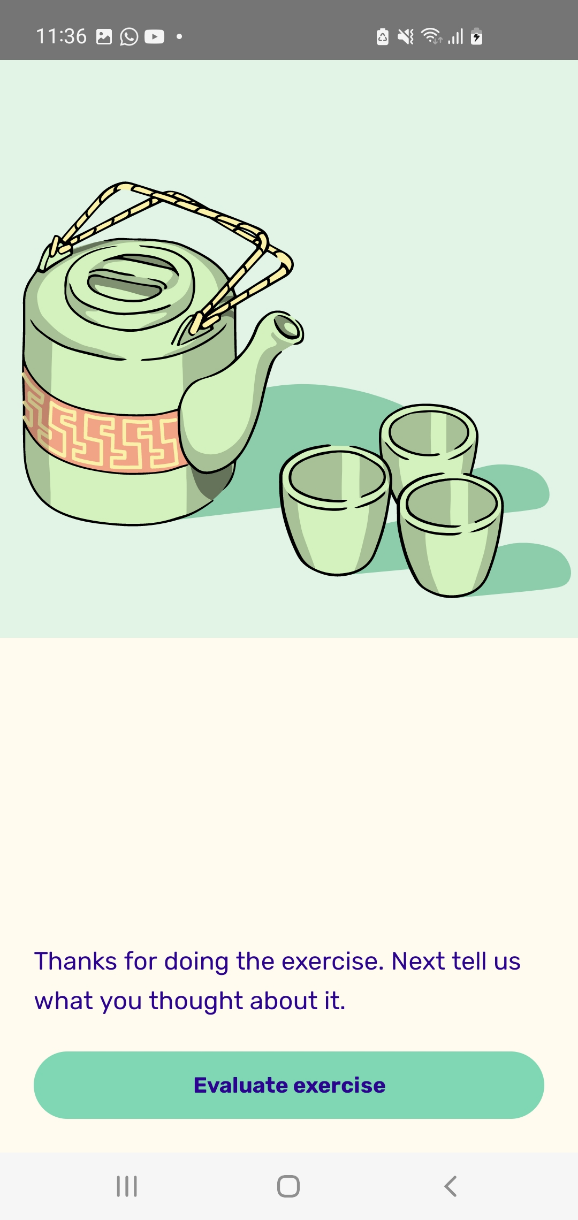


**
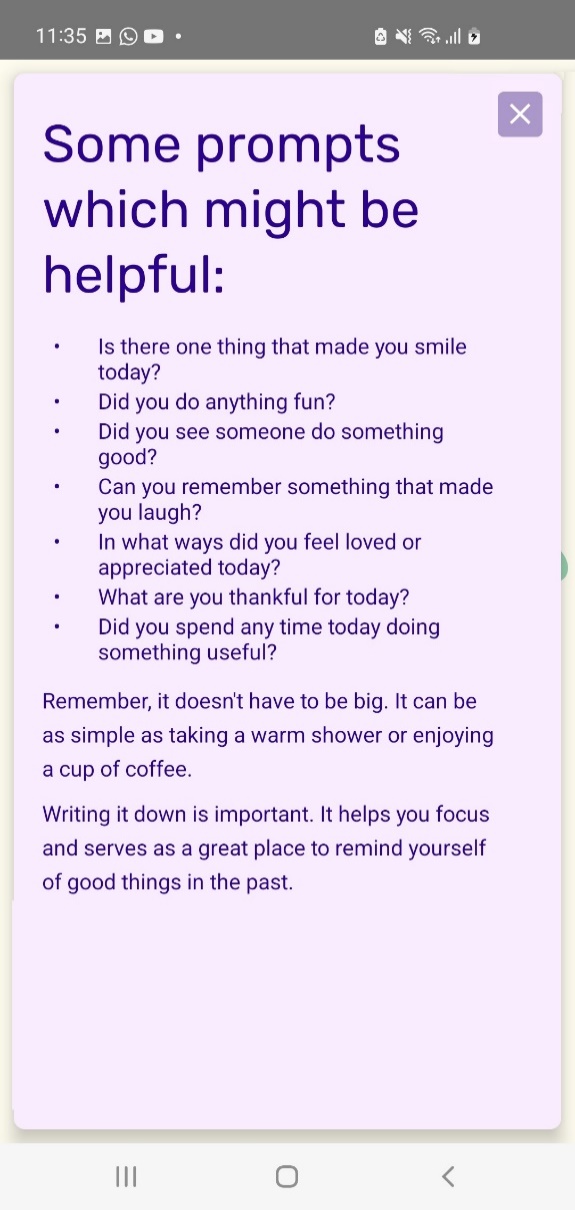
**

**Example of exercise evaluation and end page**


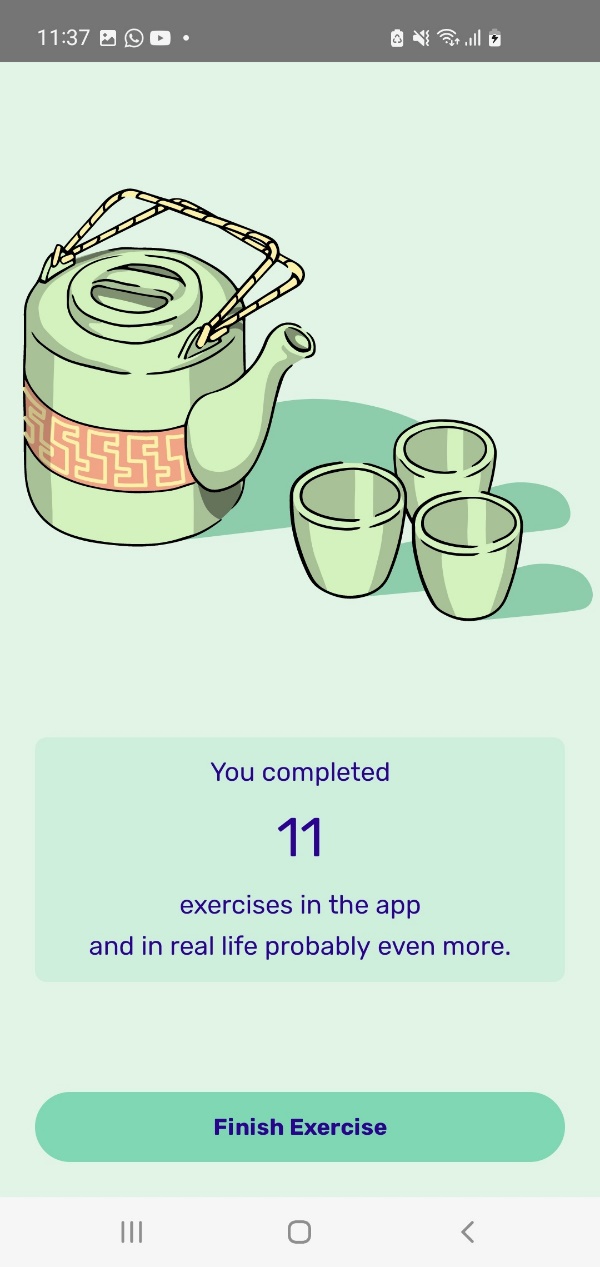

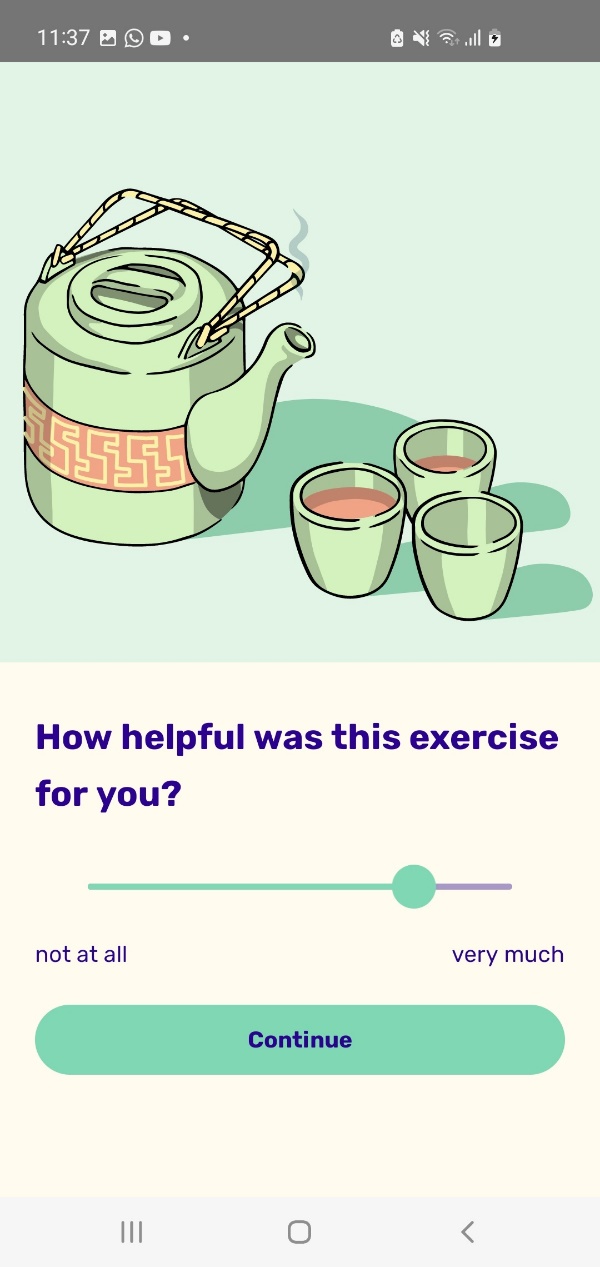

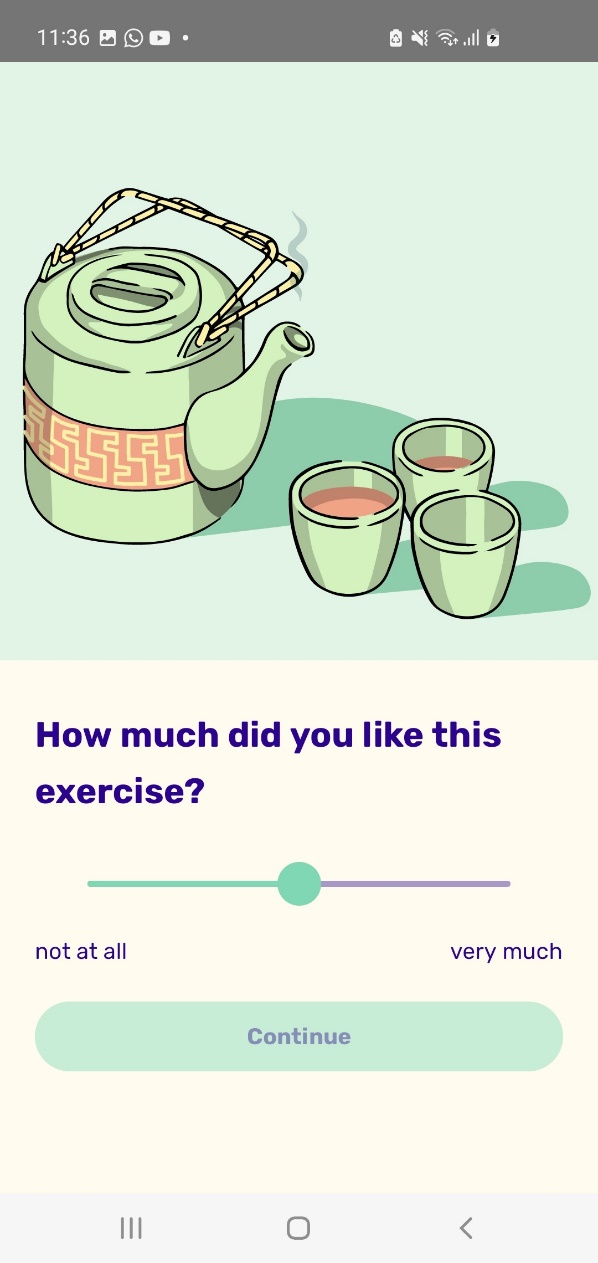


**Example of health info page**


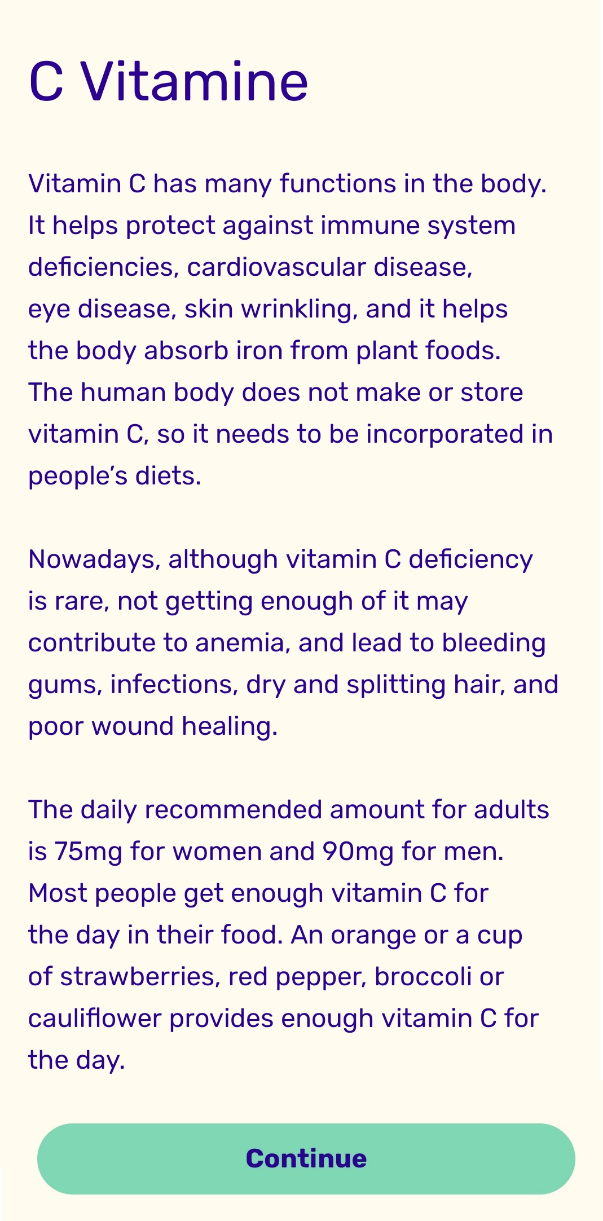

Supplement: Multimedia Appendix 1 [file resprot_v12i1e46603_app1.docx]
