# Supplementary material for: Optimization of a Transdiagnostic Mobile Emotion Regulation Intervention for University Students: Protocol for a Microrandomized Trial
Source: JMIR Res Protoc. 2023 Oct 27;12:e46603. doi: 10.2196/46603 (PMC10638637; doi:10.2196/46603)
Supplement: Multimedia Appendix 2 [file resprot_v12i1e46603_app2.docx]

**Appendix 2**

**Protocol: Semi-structured interview**

The focus of the semi-structured interview is on user experience with the intervention content, participants’ engagement patterns and system usability. During the interviews, the MIRO board will be used to refresh participants' memory of the exercises they engaged in during the study. The interviewees will have access to the wire-frames of each exercise while reporting on their experience with it.

The interview will begin with broader questions about the intervention as a whole and later shift on to the intervention categories and the separate exercises. This will allow for a general evaluation of user experience with the intervention and their engagement patterns, as well as insights into how well participants understand the different concepts included the intervention (i.e., Cognitive defusion, Self-compassion, Mindfulness, Breathing and Relaxation, Upregulation of positive affect), their preferences, and their experiences with the exercises. Additional questions might be added based on the preliminary analyses of the objective engagement outcomes (e.g., completion rates), and exercise likeability and helpfulness scores, to better explain why some exercises were preferred over others. This information will help with further optimization of the system.

**Table S1.**

Overview of the questions used in the semi-structured interviews

| Topic | Questions |
| --- | --- |
| General experience with the intervention | - What do you think about the app/intervention you were asked to engage with during this study?; - What made you motivated to engage with it?; - What kind of difficulties did you experience while engaging with it?, - In what setting and at what times did you usually engage with the app? Why was that? |
| Understanding different approaches | - What do you think of the articles in the app explaining different approaches that accompanied the exercises? (Did you manage to read them? Did they help you understand the exercises you were asked to complete, and how?) - Could you tell me in your own words what the different approaches you got to know during the study are about? |
| EMA and exercises (general) | - What do you think about the pre and post-assessments of how you feel at the moment? - What did you learn from it? How helpful do you find them? - What is your experience with the exercises? - What do you think about the set up of the exercises (the length, instructions clarity, the steps you have to take to complete them, the visualization, interactivity) |
| Intervention categories *Note. Each intervention category will be looked at separately*. | - What do you think about the information that was provided about the exercise? (Was it enough for you to understand and to complete the exercise? If not, what was missing?) - Which type of exercises did you connect with / like  the most? - Could you point out/ name exercises that you find the most helpful? Why is that? - Could you point out/name exercises that you find the least helpful? Why is that? - Could you describe how you felt while and after doing an exercise? - Which techniques did you or you think you will use in your everyday life? |
| Other | Additional questions may be derived from engagement data analysis |
