# Supplementary material for: Optimization of a Transdiagnostic Mobile Emotion Regulation Intervention for University Students: Protocol for a Microrandomized Trial
Source: JMIR Res Protoc. 2023 Oct 27;12:e46603. doi: 10.2196/46603 (PMC10638637; doi:10.2196/46603)
Supplement: Multimedia Appendix 3 [file resprot_v12i1e46603_app3.docx]

## **Appendix 4**

**Adherence protocol**

The adherence protocol involves tracking completed and missed sessions (i.e., completion of pre-EMA, intervention or control intervention, and completion of post-EMA is considered as one session) using a research manager platform which may trigger 4 different types of warning. The warning type is based on the number of sessions missed. Specifically, warning type one is activated when a participant misses six consecutive or six total sessions. Warning type two is activated when participant misses ten consecutive sessions or ten total sessions. Warning type three is activated when the participant misses thirteen sessions, and the researcher has not yet reached them via a phone call. Warning type four is activated when sixteen total sessions are missed. See Table 1. with explanation of actions for each warning type.

Table 1. Adherence protocol: four warning types and the corresponding actions.

| Warning type | Number of sessions missed | Action |
| --- | --- | --- |
| 1 | 6 consecutive or total | Participant will receive an e-mail notifying them that they missed 6 sessions and reminding them a maximum of two sessions may be missed before being excluded from the lottery for a 50-euro voucher. |
| 2 | 10 consecutive or total | The researcher will contact the participant via phone to inquire about any technical issues, address questions participants might have, and verify if they still wish to continue participating in the study. |
| 3 | 13 total | If the participant has not been previously reached via phone, the researcher will make another attempt to reach them. However, if the participant cannot be reached, an e-mail will be sent to inquire about technical issues, provide an opportunity for clarifying questions, and confirm their wish to proceed with the study. |
| 4 | 16 consecutive or total | A final reminder will be sent to the participant, inviting them to report any technical issues, ask clarifying questions, or communicate their participation status. Although participants missing 16 sessions or more may continue with engaging with the intervention, no further attempts will be made to encourage their participation in the study. |
