# Supplementary material for: Optimization of a Transdiagnostic Mobile Emotion Regulation Intervention for University Students: Protocol for a Microrandomized Trial
Source: JMIR Res Protoc. 2023 Oct 27;12:e46603. doi: 10.2196/46603 (PMC10638637; doi:10.2196/46603)
Supplement: Multimedia Appendix 4 [file resprot_v12i1e46603_app4.docx]

**Appendix 4**

**The analytic method used for MRT data analysis**

This supplementary appendix describes in detail the data analysis method for the primary aim of the study: ***Optimizing a Transdiagnostic Emotion Regulation Intervention for University Students: Protocol for a Micro Randomized Trial***. We will here use the Centered and Weighted Least-Squared (WCLS) method to estimate and test causal excursion effects on proximal outcomes for analyzing data from a MRT [23] to inform the design of a JITAI, developed by Boruvka et al [105].

All analyses will be conducted using the R programming language (R Core Team [106]). To conduct the described analyses, the R package geepack will be used.

1. Notation and study set-up

- **Decision point (t):** In the current study, decision points take place every day, twice a day. One in the morning and one in the afternoon/evening. Over the course of 21 days, there are 42 decision points (t = 1, ..., T where T = 2x21 = 42).
- **Randomization probabilities (p):** The randomization probability for delivering an intervention is 60 % and 40% for delivering a control intervention. Each of the 5 intervention subcategories have an equal 12% probability to be delivered at each decision point.
- An **indicator of whether an intervention or control intervention** is delivered at the decision point t **(A_t_):** A_t_ = 1 if the participant is randomized to receive an intervention or A_t_=0 when the participant is randomized to receive a control intervention. Delivery of intervention vs. no intervention is randomized at the decision point t regardless of participant availability.
- **Intervention completion (I_t_)**: In our study, participants are assumed to be available (availability = 1) when they complete pre-EMA. Availability = 0 otherwise.  When available, participants can decide to not interact with a given intervention, implying that the marginal intervention effect is also dependent on the randomized exercises being completed. Exercise completion happens after an exercise is randomized and could be influenced by a given exercise; whereas availability status as conceptualized in standard JITAIs is a precondition assessed before randomization and determining whether randomization takes place. In our study design, exercise completion does also provide information on whether a participant is exposed to the given exercise or not, similar to what availability status conceptually implies. Therefore, in our model availability status is replaced by a term coding for whether the full intervention flow was completed at time t (I_t_ = 1) or not (I_t_ = 0).
- **Primary outcomes of interest** consist of two continuous variables, **Y_pos,t+1_**  for positive affect and **Y_neg,t+1_**  for negative affect measured after the intervention at decision point t
- **Zt:** A vector of control variables consisting of summaries formed from t and past and present observations. Specifically, Z denotes the observations from EMA measured before participants complete the exercise to control for noise, consisting of positive (pos) and negative (neg) affect scores. These variables are expected to correlate with the primary outcomes of interest (Y_pos_,_t+1_ , Y_neg,t+1_), and will therefore be included in the analyses as control variables to reduce the noise
- d_t_: Day in the study coded as 0, 1, 2, 3 …, 20
- X_t,5_: Intervention category to which each randomized exercise: (a) upregulation of positive affect, (b) mindfulness, (c) cognitive defusion, (d) relaxation and breathing, and (e) self-compassion.

2 The Causal Effects

Following a decision point (i.e., every morning and evening), participants are asked to complete an EMA and are randomized either to an intervention (60% likelihood) or to a control intervention (40%).

**Research goal 1:** We are interested in the average causal excursion effect on participants’ emotional state(s) as a result of engaging in an intervention. That is the expected value of the contrast in emotional state(s) following a decision point t when the intervention were delivered at t versus when the intervention were not delivered at t [potential outcomes]. To reduce noise we will condition on the results of pre-EMA (Z_t_).

The analyses for the two outcomes (Y_pos, t+1_ and Y_neg,_) will be conducted separately. To control for multiple comparisons, Bonferroni correction will be used (ie., we will divide the by the number of comparisons being made (i.e., 2, hence α = .025).

The estimated effect, averaged over time in study and availability, of completing an intervention vs a control intervention is given by β_0_ in the below model for **Y_pos,t+1_** and **Y_pos,t+1_** , respectively (Klasnja et al [51]):

α_0_ + α_1_ Z_t_ + β_0_ (A_t_ - 0.6)

Note that this model is fit across all the available decision points (i.e., for cases when participants engaged in the prompted intervention, meaning that the I_t_=1).

- Yt+1 represents the emotional state of participants as an outcome of engaging in the intervention. The *pos* and *neg* notations represent two analyses as we will conduct analyses for the negative and positive affect individually.
- β_0_ indicates the average causal excursion effect, marginal over all unobserved and observed variables at times up to and including t, and marginal over time, of intervention vs. control intervention on participants’ emotional state.
- At indicates whether the intervention (1) or control intervention (0) was randomized to the participant, and it is standardized by subtracting the mean treatment rate of 0.6.
- α_0_ +  α_1_Z_t_ is used to reduce the residual variance in Yt+1. Z_t_ in this analysis represents the value of EMA at decision point t before participants complete the exercise.

**Research goal 2:**

The estimated time varying effect, averaged over time in study and availability, of completing an intervention vs a control intervention (decrease or increase) is given by β_1_ in the below model for **Y_pos,t+1_** and **Y_pos,t+1_** , respectively (Klasnja et al [51]):

 α_0_ +  α_1_Z_t_ + β_0_ (A_t_ - 0.6) + β_1_ (A_t_ - 0.6)d_t_

- Ypos, t+1 and Yneg, t+1 represent the emotional state of participants as an outcome of engaging in the intervention. The *pos* and *neg* notations represent two analyses as we will conduct analyses for the negative and positive affect individually.
- d_t_ in this model represents the day of the study and is denoted as: 0, 1, …, 20.
- The coefficient β_0_ represents the initial effect of the intervention on the participants’ positive / negative affectivity.
- The coefficient β_1_ describes how the effect of the intervention on participants’ positive/ negative affect scores changes for each additional day in the study.
- α_0_ + α_1_Z_t_ is used to reduce the residual variance in Yt+1. Zt in this analysis represents the value of EMA (emotional states; _positive_ and _negative_) measured before participants complete the exercise used to control for noise.

**Subgoal:** We are also interested in the average causal excursion effect of the different components of the intervention (i.e., different intervention types: upregulation of positive affect, mindfulness, cognitive defusion, relaxation and breathing, and self-compassion) vs. control intervention on participants’ emotional state(s).  Conceptual analysis for the effect of separate intervention types is as follows:

 Y_pos, t+1_ and Y_neg, t+1_ ”∼” α_0_ +  α_1_Z_t_ + β_0_ (A_1t_ - 0.12) + β_1_ (A_2t_ - 0.12) + β_2_ (A_3t_ - 0.12) + β_3_ (A_4t_ - 0.12) + β_4_ (A_5t_ - 0.12)

The difference between this and the first conceptual model (See research goal 1) is that we added the intervention categories into the equation (A(1-5)t). The probability for an intervention to be randomized is 60% (p = .6), but the probability for a separate intervention category type to be randomized is 12% (p = .12)

- β _0,1,2,3,4_ indicate the average causal excursion effect, marginal over all unobserved and observed variables at times up to and including t, and marginal over time, of each intervention category vs. control intervention on participants’ emotional state.

Note that A_t_ in the equation are coded as specified below:

- A_1t_= 1 if a participant is assigned the upregulation of positive affect exercise at the t^th^ decision point; A_1t_ = 0 if otherwise.
- A_2t_ = 1 if a participant is assigned the mindfulness exercise at the t^th^ decision point; A_2t_ = 0 if otherwise.
- A_3t_= 1 if a participant is assigned the cognitive defusion exercise at the t^th^ decision point; A_3t_ = 0 if otherwise.
- A_4t_ = 1 if a participant is assigned the breathing and relaxation exercise at the t^th^ decision point; A_4t_ = 0 if otherwise.
- A_5t_= 1 if a participant is assigned the self-compassion exercise at the t^th^ decision point; A_5t_= 0 if otherwise.
